# Green synthesis of thiourea derivatives from nitrobenzenes using Ni nanoparticles immobilized on triazine-aminopyridine-modified MIL-101(Cr) MOF

**DOI:** 10.1038/s41598-023-40190-w

**Published:** 2023-08-10

**Authors:** Sara Heidari, Sedigheh Alavinia, Ramin Ghorbani-Vaghei

**Affiliations:** Department of Organic Chemistry, Faculty of Chemistry, Bu-Ali Sina University, Hamadan, 6517838683 Iran

**Keywords:** Catalysis, Green chemistry, Materials chemistry, Organic chemistry

## Abstract

Nanohybrid metal–organic frameworks (MOF) have recently been considered next-generation catalysts regarding their unique features like large surface-to-volume ratio, tailorable geometry, uniform pore sizes, and homogeneous distribution of active sites. In this report, we address the triazine-aminopyridine-modified 3D Cr-centred MOF MIL-101(Cr)-NH_2_ following a post-synthetic modification approach. The excellent chelating ability of triazine-aminopyridine was applied to immobilize Ni ions over the host matrix MOF. The as-synthesized material was physicochemically characterized using various analytical techniques like FT-IR, electron microscopy, EDS, elemental mapping, XRD, and ICP-OES. Subsequently, the material has been catalytically employed in synthesizing new thiourea derivatives by reacting to nitrobenzene derivatives and phenyl isocyanate. The catalyst was isolated by centrifugation and recycled in 6 consecutive runs without momentous loss of its reactivity.

## Introduction

Numerous studies have been done since MOFs appeared in our world nearly two decades ago, and new studies continue to be done with increasing interest^1–6^. Properties such as large surface areas, a highly porous structure, and easy functionalization can be counted among the important features of MOFs that deserve this attention^7–9^. They have many cutting-edge applications like storing gas, catalyzing processes, delivering drugs, encapsulating material, supercapacitors, and heavy metal absorbents^10–13^.

Compared to other classes of porous materials, MOFs exhibit greater durability, diverse morphologies, and different porosity. MOFs among the porous compounds are stable under various conditions and can maintain porosity due to their chemical and thermal resilience^14–17^. Due to the wide applications of MOF in science and technology, it is of great interest to create new molecular scaffolds with different structures to improve their capacity and selectivity^18–23^. Although MOFs have great potential as heterogeneous catalysts and have attracted great interest from researchers, plans to use them at the industrial stage have yet to make significant progress^13,24,25^. Confinement of the active species within the pores can provide the catalyst with some degree of protection from other reactive species that is difficult to achieve in homogeneous phases through ligand manipulation alone^26,27^.

Post-synthetic modification (PSM) through inserting various organic and inorganic functionalities into the framework plays a significant role in optimizing the chemical and physical properties of organic supports such as magnetic nanoparticles, silicates, boehmite, and MOFs^2,13,28–36^. A large variety of supported nanostructured catalysts based on MOFs have been reported during the past decade, highlighting the critical role of such materials in developing novel catalytic materials with high selectivity^37^. One of the most important challenges in catalytic processes is investigating the applications of post-synthetic MOF processes and the formation of organometallic complexes for use in organic synthesis^38^.

**Figure 1:**
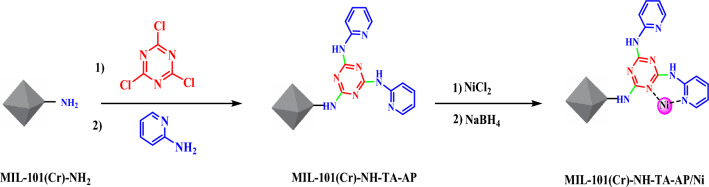
Preparation of MIL-101(Cr)-NH-TA-AP/Ni nanocatalyst.

Thiourea derivatives play a vital role in catalyst modification and synthesis of intermediates and natural products^39,40^. Based on this importance, studies involving new catalyst systems and methodologies continue attracting attention. Different methods for the preparation of thiourea derivatives have been developed. In 2014, Nguyen and colleagues investigated the synthesis of thiourea derivatives from the reaction of isocyanides with aliphatic amines in the presence of elemental sulfur. In another report, a condensation between amines and carbon disulfide in an aqueous medium allows efficient synthesis of symmetrical and unsymmetrical substituted thiourea derivatives. The synthesis of thiocarbonyl from the combination of sulfur and chloroform in a two-step process was reported by Tan in 2017. A dichloromethane-mediated reaction of carbamoyl isothiocyanates with amines was reported by Linton (SI, Fig. 1, Eqs. 1–4)^41–44^. Most of the hitherto known methods suffer from limitations such as harsh reaction conditions, and use of expensive, toxic catalysts, the formation of side products, and poor yields of desired products. Regarding the important biological properties of synthetic thiourea compounds, an effective procedure for fabricating thiourea derivatives was developed through nitroarene reduction using functionalized MOF (SI, Fig. 1, Eq. 5).

Because nitrobenzenes are toxic, they have adverse effects on humans and other organisms and are a pollutant in the environment^45^. Therefore, developing new catalytic systems for effective and economical removal methods is of great importance. Besides, reducing nitrobenzenes to synthesize thiourea derivatives is a quietly significant synthon in organic synthesis and is present in various pesticides, pharmaceuticals, and fine chemicals^46–48^. Despite developing heterogeneous catalysts with metals such as Pd, Au, Pt, and Mo, selective reduction of nitro compounds is still challenging under mild conditions^47^.

Regarding the mentioned points, the present article introduces the design and characterization of a new Cr-based MOF functionalized triazine-aminopyridine via post-synthetic modifications. Triazine-aminopyridine is a recognized and excellent chelating ligand, and we exploited it to anchor Ni ions at the outer shell of MIL-101(Cr)-NH_2_. Ni NPs species were then successfully decorated on prepared support (MIL-101(Cr)-NH-TA-AP) (Fig. 1). The catalytic activities of this successfully synthesized nanocatalyst were tested in synthesizing thiourea derivatives through nitrobenzene reduction. Operational simplicity, green reaction conditions, simple and inexpensive procedure, high efficiency, short reaction times, easy catalyst separation, and reusability for several consecutive cycles are the key advantages of this protocol. The other key advantage of this protocol is that the reactions were found to be complete at room temperature. This method is found to tolerate substrates having electron-donating and withdrawing functionalities. In addition to the above advantages, we also observed this transformation is equally effective in water as a green solvent.

## Experimental

### Synthesis of MIL101 (Cr)-NH_2_

MIL101 (Cr)-NH_2_ was synthesized using solvothermal methods^49^.

### Synthesis of triazine-aminopyridine-modified MIL-101(Cr)-NH_2_ MOF (MIL-101(Cr)-NH-TA-AP)

A solution of cyanuric chloride (0.75 g; 4 mmol), MIL-101(Cr)-NH_2_ (0.5 g), and DMF (10 mL) was stirred at ambient temperature for 12 h. In the next step, 2-aminopyridine (0.94 g; 8 mmol) was added and stirred for 12 h. The obtained residue was filtered and washed with ethanol (2 × 10 mL) and dried in a vacuum desiccator to obtain MIL-101(Cr)-NH-TA-AP as a stable powder.

### Synthesis of MIL-101(Cr)-NH-TA-AP/Ni nanocomposite

Nickel nanoparticles were immobilized on the surface of the synthesized MIL-101(Cr)-NH-TA-AP according to the previous methods (Fig. 1).

### General method for the fabrication of thiourea derivatives

A mixture of nitrobenzene analogs (1 mmol), sodium borohydride (1.5 mmol), phenyl isocyanate (1 mmol), and MIL-101(Cr)-NH-TA-AP/Ni (50 mg, 0.1 mol%) was stirred at room temperature in H_2_O (2 mL) for the suitable time, as illustrated in Table 2. After the end of the reaction, the catalyst was filtered off. The residue was extracted with ethyl acetate to obtain pure products in 91–98% yields.

## Results and discussion

**Figure 2:**
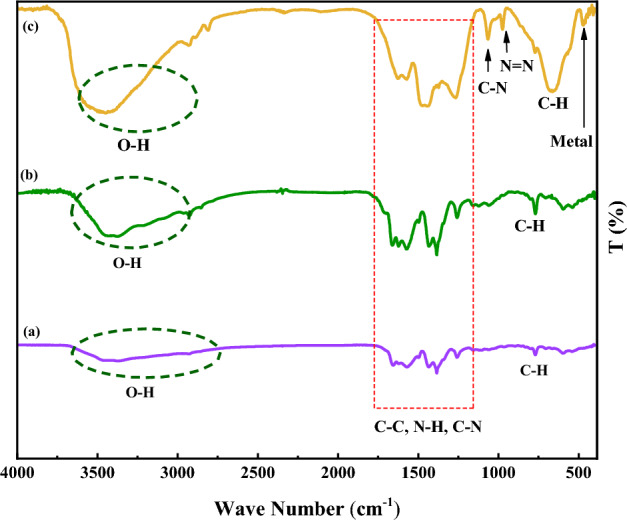
FT-IR spectra of (**a**) MIL-101(Cr)-NH_2_, (**b**) MIL-101(Cr)-NH-TA-AP, and (**c**) MIL-101(Cr)-NH-TA-AP/Ni.

Figure 2 illustrates the FT-IR absorption spectra of MIL-101(Cr)-NH_2_, MIL-101(Cr)-NH-TA-AP, and MIL-101(Cr)-NH-TA-AP/Ni. The stretching vibrations at 3400 cm^−1^ indicate the symmetric modes of the N–H bonds attached to the Cr atoms (Fig. 2a)^50^. Comparing spectra of MIL-101(Cr)-NH-TA-AP and MIL-101(Cr)-NH-TA-AP/Ni (Fig. 2b,c with Fig. 2a) confirmed the successful functionalization. Finally, considering the MIL-101(Cr)-NH-TA-AP/Ni catalyst spectra, the C=N vibration shifts around 1663 cm^-1^ due to the interaction between MIL-101(Cr)-NH-TA-AP and Ni NPs (1685 vs. 1663 cm^−1^). Moreover, the bending vibration of NH_2_ in the MIL-101(Cr)-NH-TA-AP/Ni shifts around 3338 cm^-1^ (3422 vs. 3338 cm^−1^).

**Figure 3:**
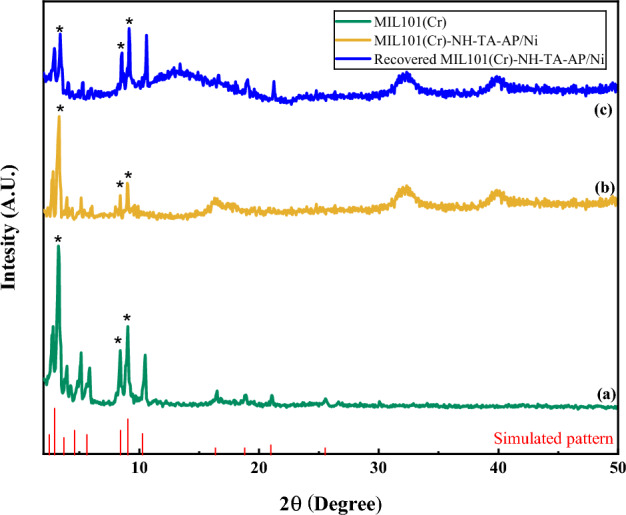
XRD pattern of MIL-101(Cr) (**a**), MIL-101(Cr)-NH-TA-AP/Ni (**b**), and recovered MIL-101(Cr)-NH-TA-AP/Ni (**c**).

XRD analysis was used to check the crystal structure of MIL-101(Cr)-NH_2_ and MIL-101(Cr)-NH-TA-AP/Ni samples (Fig. 3). The major diffraction peaks of MIL-101(Cr)-NH_2_ appeared at 2θ angles of 3.25°, 8.41°, 9.02°, 10.5°, and 16.49°, which are in agreement with pattern reported in the literature (Fig. 3a)^49,50^. In the XRD spectrum of MIL-101(Cr)-NH-TA-AP/Ni, a minor shift in position and width, few intense peaks, and several sharp peaks in the pattern are observed, confirming the successful formation of the mentioned nanocatalyst (Fig. 3b). Moreover, the presence of sharp peaks in 2θ between 10° and 50° suggests the successful functionalization of MIL-101(Cr)-NH_2_. Afterward, using Scherrer and Bragg’s equations, the average monthly distance and crystal size was determined to be 40 nm. These results are in good agreement with the result obtained from SEM images. Figure 3c illustrates XRD patterns of recycled MIL-101(Cr)-NH-TA-AP/Ni nanocomposite. As clearly can be seen from the XRD image, the catalyst retains its initial crystallinity and particles size (Fig. 3c). Since post-synthetic modification of MIL-101(Cr)-NH_2_ leads to the synthesis of the final catalyst with less crystallinity, and the simulated pattern is only used for single crystal materials, in this research, the simulated pattern of MIL-101(Cr)-NH_2_ was added to Fig. 3. In the functionalized MOF, due to the presence of functional groups, the triazine-aminopyridine groups occupy the available surface of the MOF. As a result, some peaks are removed, which is consistent with previous studies^51^.

**Figure 4:**
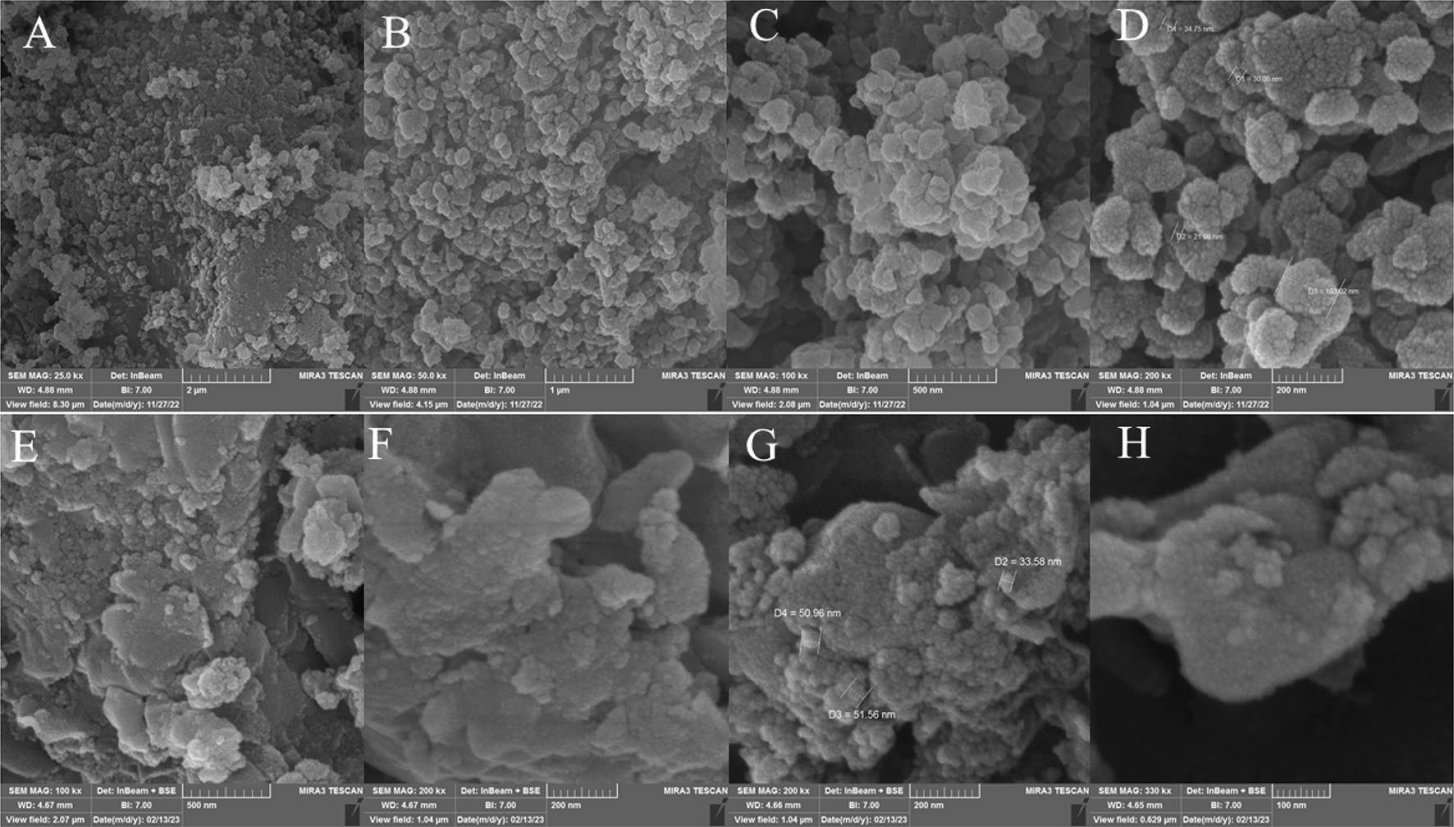
FESEM images of MIL-101(Cr)-NH_2_ (**A**–**D**) and MIL-101(Cr)-NH-TA-AP/Ni (**E**–**H**).

SEM analysis was applied to examine the morphological and chemical changes of MIL-101(Cr)-NH-TA-AP/Ni. The SEM image of the MIL-101(Cr)-NH indicates almost a spherical structure morphology. In addition, according to Fig. 4A–D, there is no significant change even after the immobilization of metal species. It is of note that functionalized MOF can retain Ni NPs species in the pores and prevent their agglomeration (Fig. 4E–H).

**Figure 5:**
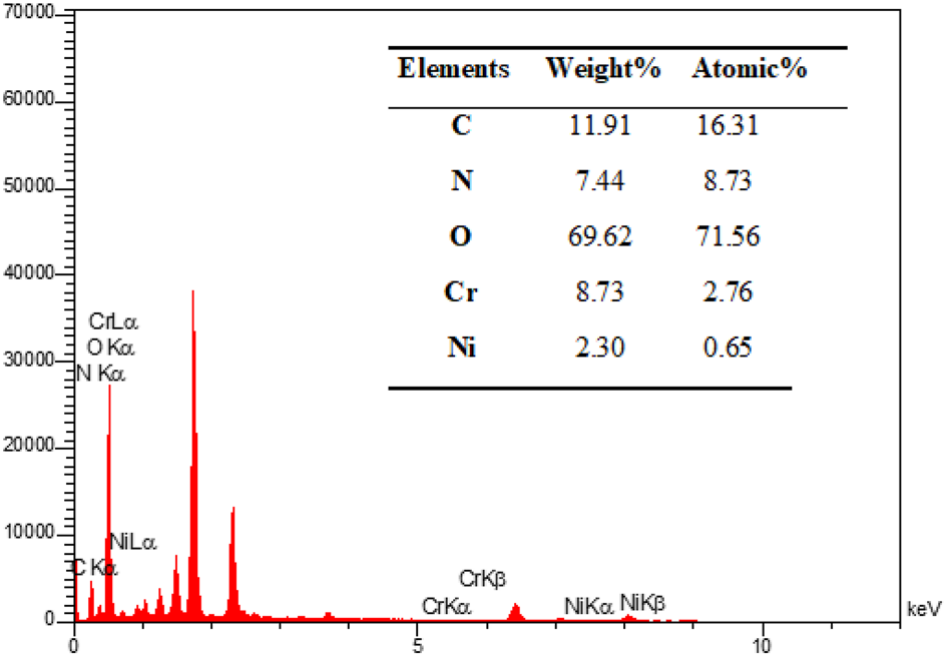
EDX spectrum of MIL-101(Cr)-NH-TA-AP/Ni.

**Figure 6:**
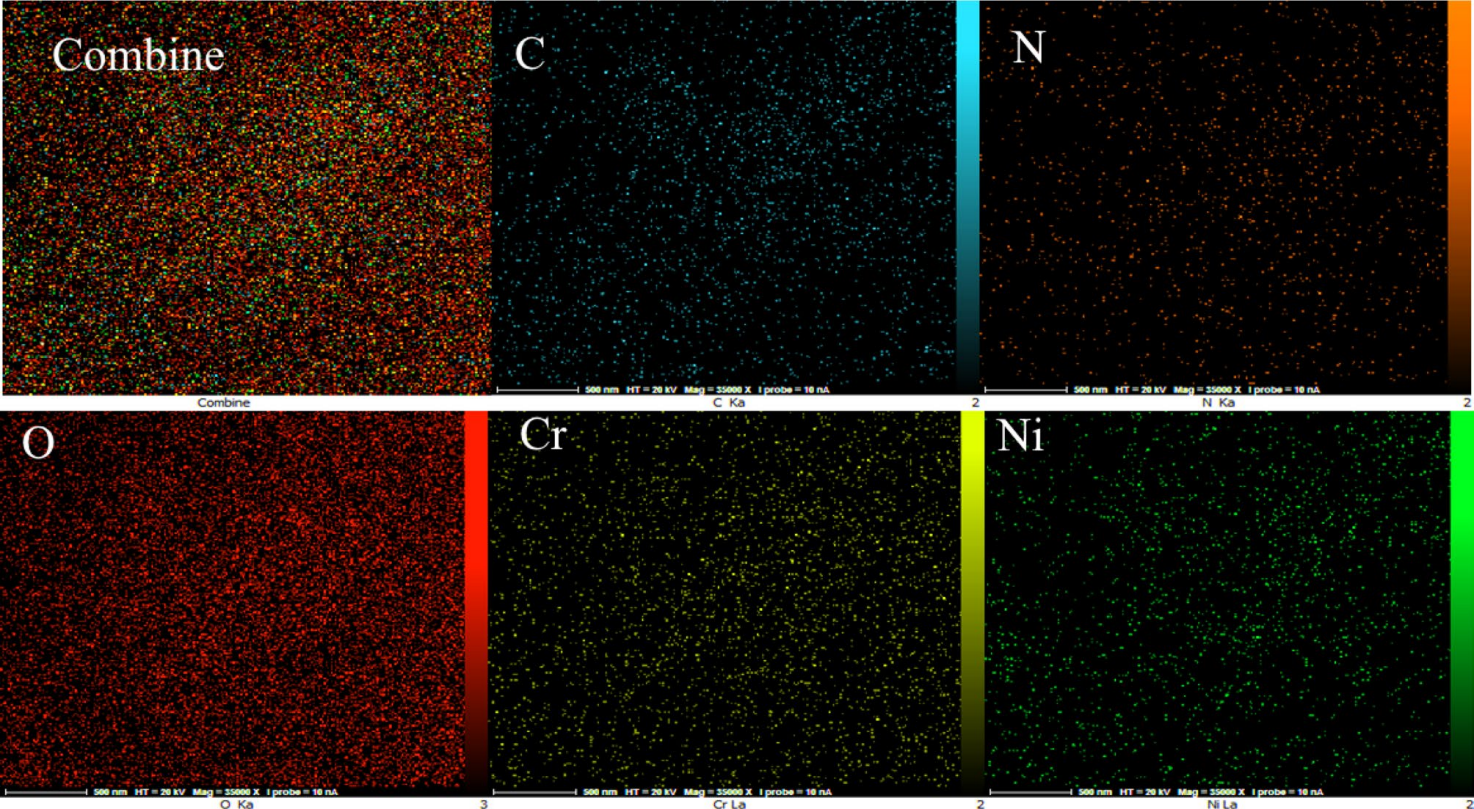
Elemental mapping of MIL-101(Cr)-NH-TA-AP/Ni.

The elemental analysis of the present MIL-101(Cr)-NH-TA-AP/Ni catalyst is presented in Fig. 5. The elemental mapping of MIL-101(Cr)-NH-TA-AP/Ni nanocatalyst is depicted in Fig. 6. As can be seen, Cr, N, C, Ni, and O elements are present in the structure of nanocatalyst with a uniform distribution.

**Figure 7:**
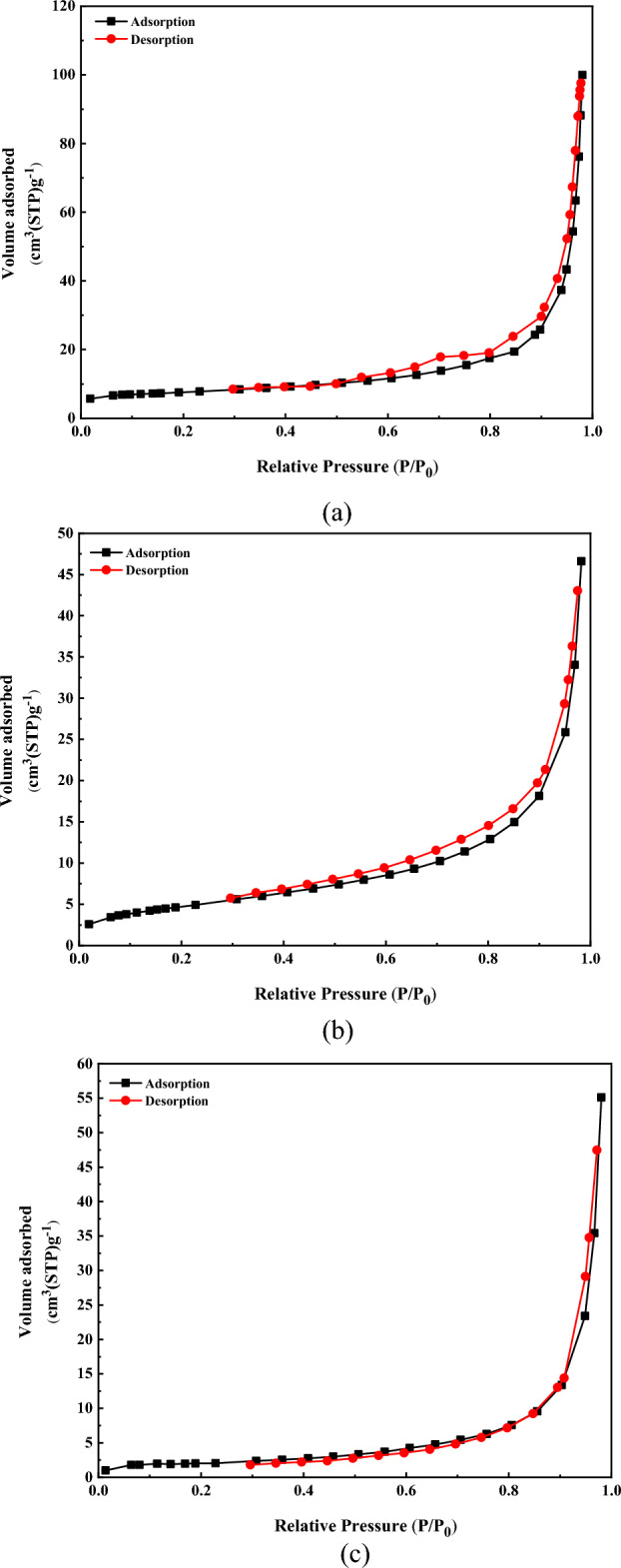
N_2_ adsorption isotherms by the BET analysis of MIL-101(Cr)-NH-TA-AP (**a**), MIL-101(Cr)-NH-TA-AP/Ni (**b**), and Recovered MIL-101(Cr)-NH-TA-AP/Ni (**c**).

Nitrogen adsorption–desorption isotherm measurement of MIL-101(Cr)-NH-TA-AP was undertaken to confirm the porous nature of the catalyst and estimate their surface area (Fig. 7a). The analysis of the BET revealed the formation of nanoparticles with a total pore volume of 0.15 cm^3^/g, an average pore diameter of 10.61 nm, and a surface area of 22.04 m^2^/g (Table 1). The adsorption isotherm is type IV, and the presence of mesoporous structure in the material is evident from the appearance of the hysteresis loop. From this BET analysis, it is inferred that our nano-support has a mesoporous nature surface and huge surface area, which is a primary property of excellent catalysts. Figure 7b shows the N_2_ adsorption and desorption isotherms of the MIL-101(Cr)-NH-TA-AP/Ni. These isotherms exhibit type V isotherms (typical of mesopores) and type H3 hysteresis loops (indicating slit-shaped pores). According to the Langmuir adsorption isotherm, the specific surface area of the MIL-101(Cr)-NH-TA-AP/Ni is 17.16 m^2^g^−1^. Figure 7c presents the N_2_ adsorption and desorption isotherms of the recovered MIL-101(Cr)-NH-TA-AP/Ni. They indicate type V isotherms (typical of mesopores) and type H3 hysteresis loops (indicating slit-shaped pores). According to the Langmuir adsorption isotherm, the specific surface area of the recovered MIL-101(Cr)-NH-TA-AP/Ni is 7.36 m^2^g^−1^. The changes associated with the textural properties of the 6th reused catalyst can be due to the distribution of the reactants inside the pores (Table 1).

**Table 1:** Results of the Langmuir and BET measurements of MIL-101(Cr)-NH-TA-AP, MIL-101(Cr)-NH-TA-AP/Ni, and Recovered MIL-101(Cr)-NH-TA-AP/Ni.

Parameter	MIL-101(Cr)-NH-TA-AP	MIL-101(Cr)-NH-TA-AP/Ni	Recovered MIL-101(Cr)-NH-TA-AP/Ni
a_s_ (m^2^/g)	32.97	17.16	7.36
V_m_ (cm^3^(STP) g^−1^)	7.57	3.95	1.69
V_p_ (cm^3^g^−1^)	0.15	0.072	0.085
r_p_ (nm)	10.61	1.63	12.24
a_p_ (m^2^/g)	22.04	18.73	10.83

**Figure 8:**
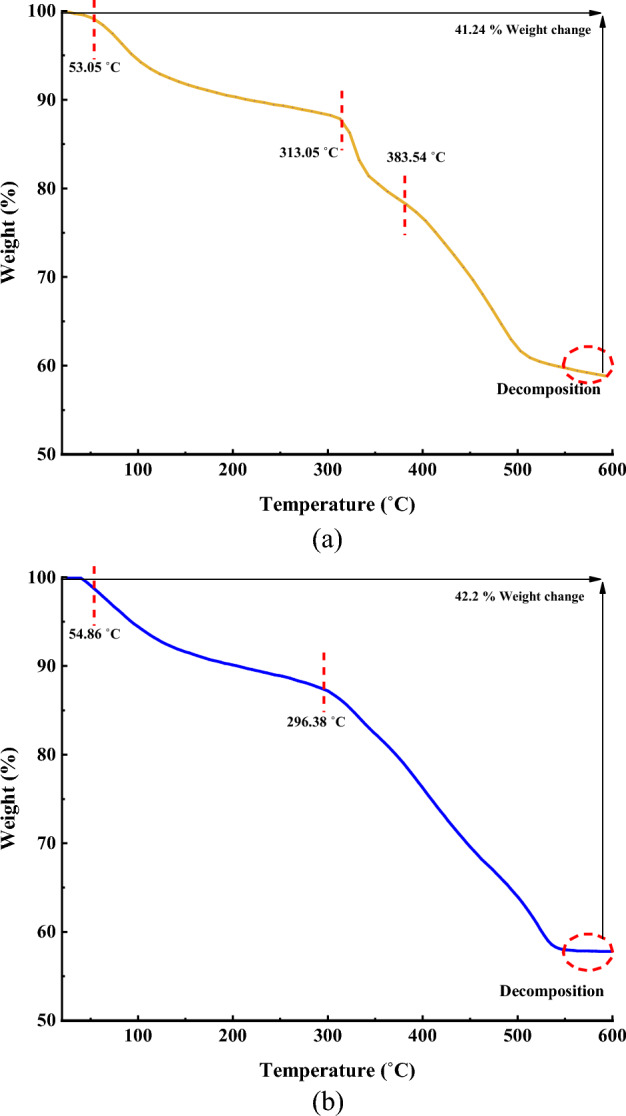
TGA curve of (**a**) MIL-101(Cr)-NH-TA-AP/Ni and (**b**) MIL-101(Cr)-NH-TA-AP/Ni after recycling.

Figure 8 shows the TGA curves showing the residual masses of MIL-101(Cr)-NH-TA-AP/Ni in the temperature range of 25 to 600 °C. The TGA curve initially displays an imperceptible weight loss of 17% in the region 90–180 °C, which verifies the loss of solvent absorbed at the surface of MIL-101(Cr)-NH-TA-AP/Ni ^52^. A significant weight loss of 35% in the 200–600 °C range can be explained by the decomposition of the immobilized ligand on the surface of MIL-101(Cr)-NH_2_. Thermal characterization data for MIL-101(Cr)-NH-TA-AP/Ni display that the catalyst was stable up to 220 °C. With increasing temperature up to 450 °C, due to the decomposition of the framework, a considerable decrease occurred in the thermal stability of MIL-101(Cr)-NH-TA-AP/Ni (Fig. 8a). The thermal and behavioral stability of the recovered MIL-101(Cr)-NH-TA-AP/Ni were studied by thermal gravimetric (TG) technique. TGA of the recovered catalyst after the 6th recycle run is also stable over a wide temperature range of 25–600 °C (Fig. 8b).

### Optimization conditions

The optimum conditions were selected by studying the effect of solvent on the reaction rate for the preparation of 1,3-diphenyl thiourea by condensation of nitrobenzene and phenyl isocyanate in the presence of 0.1 mol% (MIL-101(Cr)-NH-TA-AP/Ni) under different solvents (e.g., ethanol, methanol, EtOH:H_2_O, toluene, and H_2_O) at 50℃ (Table 2, entry 1–5). Overall, the best results were obtained with 0.1 mol% MIL-101(Cr)-NH-TA-AP/Ni using water solvent (Table 2, entry 5). Moreover, the study set of experiments determines the optimal amount of catalyst MIL-101(Cr)-NH-TA-AP/Ni require for the reaction. The reaction was carried out by variable amounts of catalyst (Table 2, entries 6–7), and maximum yield was found with 0.1 mol% of the catalyst. Further increasing the amount of catalyst MIL-101(Cr)-NH-TA-AP/Ni in the mentioned reaction condition did not significantly improve product yields (Table 2, entry 7). Examining the model reaction at room temperature demonstrated that temperature is essential to the reaction efficiency (Table 2, entry 8). The response of the studied model at different times showed that the highest yield and conversion were obtained at 30 min (Table 2, entry 9). Finally, the efficacy of the catalyst was evaluated by performing the sample reaction by MIL-101(Cr)-NH_2_-Ni. The results indicate the functionalized MOF provides the required product with great efficiency (Table 2, entry 10 vs. 5).

**Table 2:** Optimization of the reaction conditions using MIL-101(Cr)-NH-TA-AP/Ni.

Entry	*T* (°C)	Cat (mol%)	Solvent	Time (min.)	Yield (%)^a^	TOF(h^−1^)
**1**	50	0.10	Toluene	30	48	120
**2**	50	0.10	Methanol	30	60	600
**3**	50	0.10	EtOH	30	70	700
**4**	50	0.10	EtOH/H_2_O	30	80	800
**5**	50	0.10	H_2_O	30	98	**980**
**6**	50	0.05	H_2_O	30	67	1340
**7**	50	0.12	H_2_O	30	98	816.6
**8**	r.t	0.10	H_2_O	30	70	700
**9**	50	0.10	H_2_O	15	51	2000
**10**	50	50 mg	H_2_O	30	58	580^b^

^a^Reaction conditions: nitrobenzene (1.0 mmol), MIL-101(Cr)-NH-TA-AP/Ni, sodium borohydride (1.5 mmol), and H_2_O (2 mL).^b^ The model reaction was investigated in the presence of MIL-101(Cr)-NH_2_-Ni.

The generality of the method was demonstrated using different substituted nitrobenzenes. The reaction scope was expanded, and excellent reaction conversions were obtained with nitrobenzenes having electron-donating and electron-withdrawing groups. This finding reveals that the substituents on nitrobenzene have no noticeable effect on the reaction conversion (Table 3). In this work, new thiourea derivatives were developed innovatively (entries 2, 5, 8, and 9).

**Table 3:** Synthesis of thiourea derivatives from nitrobenzenes.

Entry	Substrate	Product	Time (min)	Yield (%)^a^	Melting point	References
**1**			30	98	140–142	152^53^
**2**			35	97	138–140	–
**3**			30	99	152–154	159–161^54^
**4**			30	96	135–136	138–139 ^55^
**5**			35	92	127–129	–
**6**			30	97	138–139	138^56^
**7**			35	95	160–162	160^57^
**8**			40	96	156–157	–
**9**			40	93	152–154	–
**10**			40	91	160–162	158^58^
**11**			25	92	173–176	171–172^59^
**12**			20	94	172–175	172–174^59^

Reaction conditions: nitrobenzene (1.0 mmol), MIL-101(Cr)-NH-TA-AP/Ni (0.1 mol%), sodium borohydride (1.5 mmol), and H_2_O (2 mL).

^b^Isolated yield.

**Figure 9:**
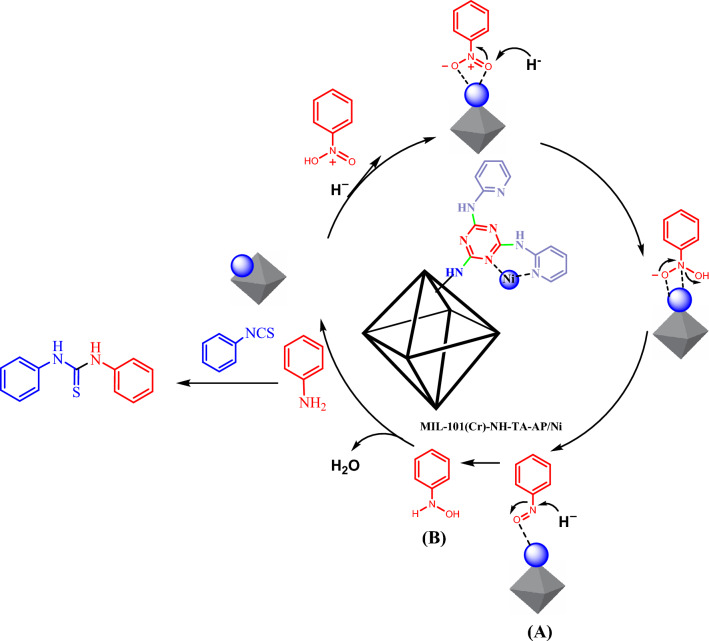
The mechanistic pathway proposed for the synthesis of thiourea from nitrobenzene derivatives.

The proposed synthesis mechanism for phenyl thiourea is illustrated in Fig. 9. The mesoporous nature of MIL-101(Cr)-NH-TA-AP/Ni catalyst gives it unique characteristics like high surface area, tunable pore size, and large pore volume, which favor their enhanced accessibility to active sites with improved mass transport/diffusion. A hydride molecule is transferred to the nitro group by adsorbing sodium borohydride on the NPs’ surface. The nitroso intermediate **A**, formed by water elimination, adsorbed on the MIL-101(Cr)-NH-TA-AP/Ni, is again reduced by the hydride transfer from sodium borohydride to the hydroxylamine B. This intermediate is further reduced to the aniline^60,61^. Lastly, phenylthiourea is obtained by the reaction between aniline and phenyl isocyanate.

**Figure 10:**
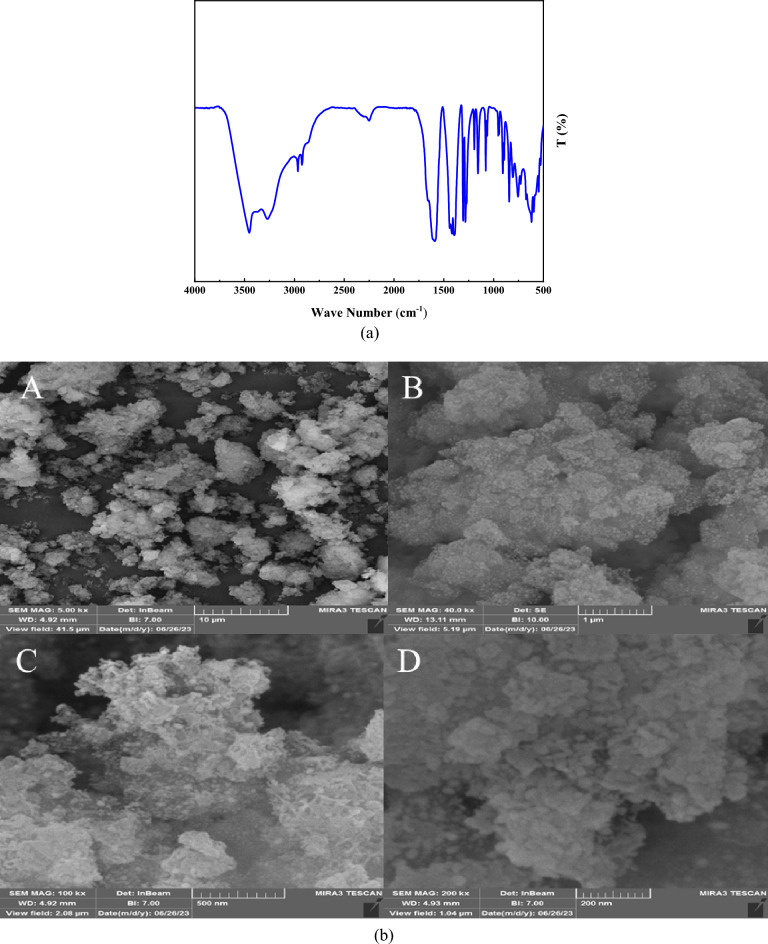

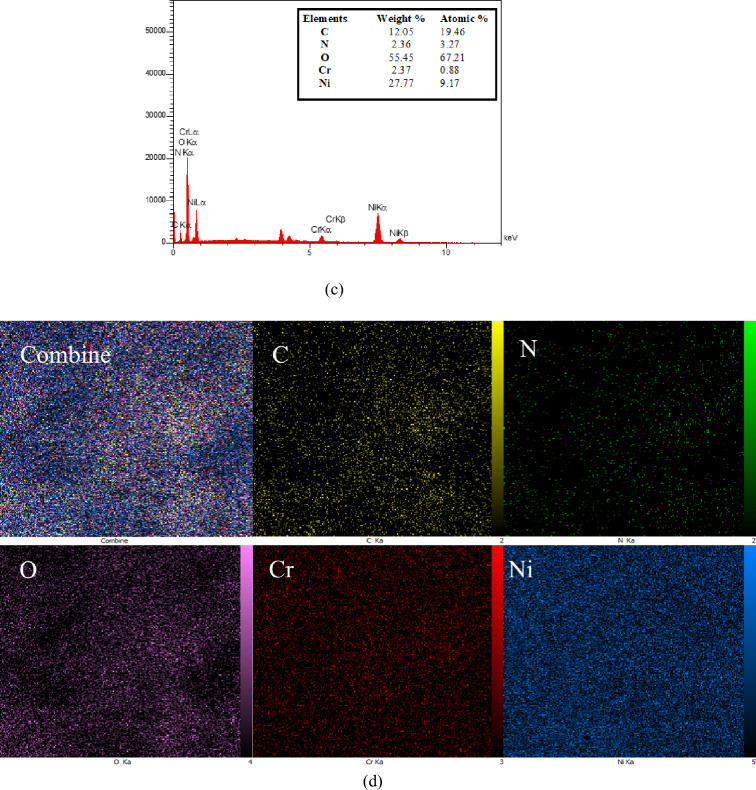
FTIR (**a**), FESEM (**b**), EDX (**c**), and elemental mapping analysis of recycled catalyst (**d**) after six runs.

**Figure 11:**
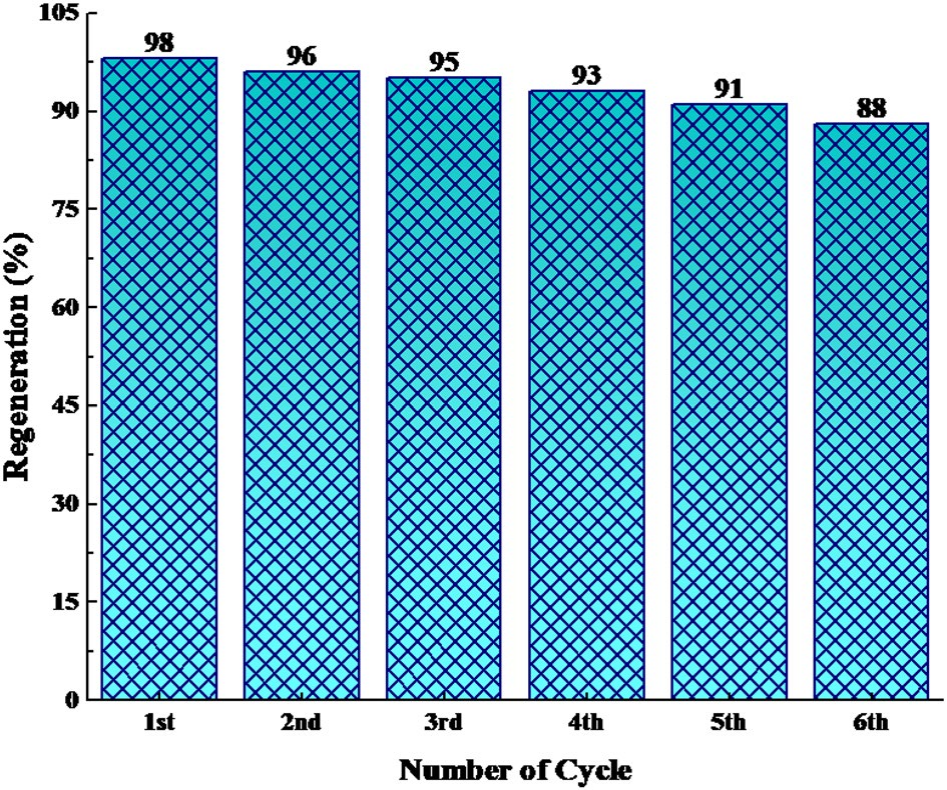
Reusability of the synthesized catalyst in the condensation of nitrobenzene and phenyl isocyanate.

The robustness of the MIL-101(Cr)-NH-TA-AP/Ni nanocomposite, as an imperative distinctiveness of catalyst in practical applications of organic transformation, was examined by successive six runs (i.e., 98, 96, 95, 93, 91, and 88). The MIL-101(Cr)-NH-TA-AP/Ni solid was recycled via simple filtration and washed with ethanol. The catalyst MIL-101(Cr)-NH-TA-AP/Ni reusability was checked in the synthesis of 1,3-diphenyl thiourea. Based on the obtained results, robust and excellent chemical stability of MIL-101(Cr)-NH-TA-AP/Ni is confirmed by FESEM, FTIR, and EDX-elemental mapping spectral analysis after six runs (Fig. 10). The FTIR of recycled catalyst confirmed the stability of the catalysts as the regeneration data do not exhibit any change after the initial patterns (Fig. 10a). In addition, FESEM of the catalyst after the 6th recycling (Fig. 10b) along with EDX-elemental mapping spectra (Fig. 10c,d) demonstrates the presence of Ni and functionalized MIL-101(Cr)-NH_2_ on the catalyst surface. The results show that the catalyst retains its initial morphology and structure without any change. As can be seen from EDX data and elemental mapping, no change occurs in elemental composition, validating the robustness of our material. The histogram in Fig. 11 represents the recyclability of the fabricated nanocatalyst for the synthesis of 1,3-diphenyl thiourea. The minor reduction observed in the catalytic ability is likely due to the normal dissipation of the catalyst in the workup process. Here, nickel concentration determined by ICP analysis changed from 8.79 mmol/g to 8.76 mmol/g after the 6th run. Therefore, the MIL-101(Cr)-NH-TA-AP/Ni nanocomposite is highly stable under the studied reaction conditions.

## Conclusion

We introduced a triazine-aminopyridine-modified MIL-101(Cr)-NH_2_ metal–organic framework (MOF) with Ni NPs decorated over its surface. Ni NPs were immobilized following a post-functionalization of triazine-aminopyridine over the core MIL-101(Cr)-NH_2_ MOF than the typical surface deposition. The excellent chelating potential of triazine-aminopyridine was exploited to deposit Ni NPs over it. The material’s structural morphology and physicochemical features were explored over different instrumental methods. In addition, atomic mapping analysis displays the uniform dispersion of active sites throughout the surface matrix. The nanocatalyst was used to synthesize thiourea derivatives through nitrobenzene reduction under mild and green conditions affording outstanding yields. The material’s robustness was validated by recycling it for 6 consecutive cycles without considerable loss of its reactivity. Moreover, Ni species had negligible leaching in the reaction medium, justifying its true heterogeneity.

### Supplementary Information

Supplementary Information.

## Data Availability

All data generated or analyzed during this study are included in this published article [and the supplementary information file].
