# Supplementary material for: Green synthesis of thiourea derivatives from nitrobenzenes using Ni nanoparticles immobilized on triazine-aminopyridine-modified MIL-101(Cr) MOF
Source: Sci Rep. 2023 Aug 10;13:12964. doi: 10.1038/s41598-023-40190-w (PMC10415257; doi:10.1038/s41598-023-40190-w)
Supplement: Supplementary file 1 — Supplementary Information. [file 41598_2023_40190_MOESM1_ESM.docx]

**Green synthesis of thiourea derivatives from nitrobenzenes using Ni nanoparticles immobilized on triazine-aminopyridine-modified MIL-101(Cr) MOF**

Sara Heidari,^a^ Sedigheh Alavinia,^a^ Ramin Ghorbani-Vaghei,*^,a^

^a^Department of Organic Chemistry, Faculty of Chemistry, Bu-Ali Sina University, 6517838683, Hamadan, Iran

Corresponding Author: Fax: Tel./Fax: +98-8138380709*

E-mail: [rgvaghei@yahoo.com &](mailto:rgvaghei@yahoo.com%20&) [ghorbani@basu.ac.ir](mailto:ghorbani@basu.ac.ir)

Contents Pages

Figure 1. Strategies for the synthesis of thiourea derivatives……………………...S3

[**Spectra data**……………………………………………………………………….S3](#_Toc97457812)

[1,3-Diphenylthiourea (3a)………………………………………………………….S3](#_Toc97457814)

1-(2,3-Dimethylphenyl)-3-phenylthiourea (3b)…………………. ……………………...S4

[1-(4-Methoxyphenyl)-3-phenylthiourea (3c)………………………………………S](#_Toc97457815)4

[1-Phenyl-3-(*p*-tolyl)thiourea (3d)…………………………………………………..S](#_Toc97457816)4

[1-(4-Ethylphenyl)-3-phenylthiourea (3e)…………………………………………..S](#_Toc97457817)4

1-Phenyl-3-(*o*-tolyl)thiourea (3f)…………………………………………… ……..S4

1-(2-Iodophenyl)-3-phenylthiourea (3g)……………………………………………S4

1-(2-Bromophenyl)-3-phenylthiourea (3h)…………………………………………S5

1-(4-Chlorophenyl)-3-phenylthiourea (3i)…………………………………………..S5

1-(4-Bromophenyl)-3-phenylthiourea (3j)…………………………………………..S5

1-(4-Fluorophenyl)-3-phenylthiourea (3k)…………………………..........................S5

1-Phenyl-3-(pyridin-2-yl)thiourea (3l)………………………………………………S5

Figure 2. FTIR Spectrum of 1,3-diphenylthiourea (3a)…………………………….S6

Figure 3.  [HNMR Spectrum of 1,3-diphenylthiourea (3a)…………………………..S6](#_Toc97457831)

[Figure 4.](#_Toc97457831) [[CNMR Spectrum of 1,3-diphenylthiourea (3a)…………………………...S](#_Toc97457831)](#_Toc97457833)[7](#_Toc97457831)

Figure 5. FTIR Spectrum of 1-(2,3-dimethylphenyl)-3-phenylthiourea (3b)……….S7

Figure 6.  [HNMR Spectrum of 1-(2,3-dimethylphenyl)-3-phenylthiourea (3b)……..S8](#_Toc97457831)

[Figure 7.](#_Toc97457831) [[CNMR Spectrum of 1-(2,3-dimethylphenyl)-3-phenylthiourea (3b)……...](#_Toc97457831)](#_Toc97457833)S8

Figure 8. FTIR Spectrum of 1-(4-methoxyphenyl)-3-phenylthiourea (3c)………....S9

Figure 9.  [HNMR Spectrum of 1-(4-methoxyphenyl)-3-phenylthiourea (3c)……….S9](#_Toc97457831)

[Figure 10.](#_Toc97457831) [[CNMR Spectrum of1-(4-methoxyphenyl)-3-phenylthiourea (3c)……...](#_Toc97457831)](#_Toc97457833)..S10

Figure 11. FTIR Spectrum of 1-phenyl-3-(*p*-tolyl)thiourea (3d)……………………S10

Figure 12.  [HNMR Spectrum of 1-phenyl-3-(](#_Toc97457831)*[p](#_Toc97457831)*[-tolyl)thiourea (3d)………………….S11](#_Toc97457831)

[Figure 13.](#_Toc97457831) [[CNMR Spectrum of 1-phenyl-3-(](#_Toc97457831)*[p](#_Toc97457831)*[-tolyl)thiourea (3d)……...](#_Toc97457831)](#_Toc97457833).................S11

Figure 14. FTIR Spectrum of 1-(4-ethylphenyl)-3-phenylthiourea (3e)…………...S12

Figure 15.  [HNMR Spectrum of 1-(4-ethylphenyl)-3-phenylthiourea (3e)…………S12](#_Toc97457831)

[Figure 16.](#_Toc97457831) [[CNMR Spectrum of 1-(4-ethylphenyl)-3-phenylthiourea (3e)……...](#_Toc97457831)](#_Toc97457833)......S13

Figure 17. FTIR Spectrum of 1-phenyl-3-(o-tolyl)thiourea (3f)…………………...S13

Figure 18.  [HNMR Spectrum of 1-phenyl-3-(o-tolyl)thiourea (3f)…………………S14](#_Toc97457831)

[Figure 19.](#_Toc97457831) [[CNMR Spectrum of 1-phenyl-3-(o-tolyl)thiourea (3f)……...](#_Toc97457831)](#_Toc97457833)..................S14

Figure 20. FTIR Spectrum of 1-(2-iodophenyl)-3-phenylthiourea (3g)……………….S15

Figure 21.  [HNMR Spectrum of 1-(2-iodophenyl)-3-phenylthiourea](#_Toc97457831)[(3g)……………..S15](#_Toc97457831)

[Figure 22.](#_Toc97457831) [[CNMR Spectrum of 1-(2-iodophenyl)-3-phenylthiourea](#_Toc97457831)[(3g)……...](#_Toc97457831)](#_Toc97457833).............S16

Figure 23. FTIR Spectrum of 1-(2-bromophenyl)-3-phenylthiourea (3h)…………..S16

Figure 24.  [HNMR Spectrum of 1-(2-bromophenyl)-3-phenylthiourea (3h)………...S17](#_Toc97457831)

[Figure 25.](#_Toc97457831) [[CNMR Spectrum of 1-(2-bromophenyl)-3-phenylthiourea (3h)……...](#_Toc97457831)](#_Toc97457833).....S17

Figure 26. FTIR Spectrum of 1-(4-chlorophenyl)-3-phenylthiourea (3i)………………S18

Figure 27.  [HNMR Spectrum of 1-(4-chlorophenyl)-3-phenylthiourea (3i)…….............S18](#_Toc97457831)

[Figure 28.](#_Toc97457831) [[CNMR Spectrum of 1-(4-chlorophenyl)-3-phenylthiourea (3i)……...](#_Toc97457831)](#_Toc97457833)...........S19

Figure 29. FTIR Spectrum of 1-(4-bromophenyl)-3-phenylthiourea (3j)………………S19

Figure 30.  [HNMR Spectrum of 1-(4-bromophenyl)-3-phenylthiourea](#_Toc97457831)[(3j)…………….S20](#_Toc97457831)

[Figure 31.](#_Toc97457831) [[CNMR Spectrum of 1-(4-bromophenyl)-3-phenylthiourea](#_Toc97457831)[(3j)……...](#_Toc97457831)](#_Toc97457833)...........S20

Figure 32. FTIR Spectrum of 1-(4-fluorophenyl)-3-phenylthiourea (3k)………………S21

Figure 33.  [HNMR Spectrum of 1-(4-fluorophenyl)-3-phenylthiourea (3k)…………….S21](#_Toc97457831)

[Figure 34.](#_Toc97457831) [[CNMR Spectrum of 1-(4-fluorophenyl)-3-phenylthiourea (3k)……...](#_Toc97457831)](#_Toc97457833)...........S22

Figure 35. FTIR Spectrum of 1-phenyl-3-(pyridin-2-yl)thiourea (3l)………………….S22

Figure 36.  [HNMR Spectrum of 1-phenyl-3-(pyridin-2-yl)thiourea (3l)………………..S23](#_Toc97457831)

[Figure 37.](#_Toc97457831) [[CNMR Spectrum of 1-phenyl-3-(pyridin-2-yl)thiourea (3l)……...](#_Toc97457831)](#_Toc97457833)................S23

Figure 1. Strategies for the synthesis of thiourea derivatives.

**Spectra data**

*1,3-Diphenylthiourea (3a)*

IR (KBr): 3207, 3119 cm^-1^, ^1^H NMR (250 MHz, DMSO) δ 9.85 (s, 2H), 7.69 (s, 1H), 7.49 (s, 3H), 7.31 (s, 4H), 7.11 (s, 2H). ^13^C NMR (63 MHz, DMSO) δ 180.07, 139.93, 128.85, 124.83, 124.04.

*1-(2,3-Dimethylphenyl)-3-phenylthiourea (3b)*

IR (KBr): 3345, 3152 cm^-1^. ^1^H NMR (250 MHz, DMSO) δ 9.58 (s, 1H), 9.41 (s, 1H), 7.49 (d, *J* = 7.7 Hz, 2H), 7.30 (t, *J* = 7.6 Hz, 2H), 7.14 – 7.03 (m, 4H), 2.25 (s, 3H), 2.12 (s, 3H). ^13^C NMR (63 MHz, DMSO) δ 181.01, 140.11, 134.16, 128.76, 128.44, 126.28, 125.82, 124.76, 124.23, 20.51, 14.64.

*1-(4-Methoxyphenyl)-3-phenylthiourea (3c)*

IR (KBr): 3213 cm^-1^. ^1^H NMR (250 MHz, DMSO) δ 9.73 (d, *J* = 8.5 Hz, 2H), 7.49 (d, *J* = 6.9 Hz, 2H), 7.32 (s, 4H), 7.10 (d, *J* = 6.2 Hz, 1H), 6.89 (d, *J* = 7.4 Hz, 2H), 3.72 (s, 3H). ^13^C NMR (63 MHz, DMSO) δ 180.32, 156.98, 140.04, 132.63, 128.81, 126.40, 124.69, 124.00, 114.10, 55.67.

*1-Phenyl-3-(p-tolyl)thiourea (3d)*

IR (KBr): 3432, 3207 cm^-1^. ^1^H NMR (250 MHz, DMSO) δ 9.77 (s, 2H), 7.48 (d, *J* = 7.7 Hz, 2H), 7.32 (dd, *J* = 13.8, 7.9 Hz, 4H), 7.12 (d, *J* = 8.1 Hz, 3H), 2.26 (s, 3H). ^13^C NMR (63 MHz, DMSO) δ 180.01, 139.98, 136.92, 133.66, 129.33, 128.82, 124.72, 124.26, 124.00, 20.96.

*1-(4-Ethylphenyl)-3-phenylthiourea (3e)*

IR (KBr): 3401, 3205 cm-1. H NMR (250 MHz, DMSO) δ 9.75 (s, 2H), 7.47 (d, *J* = 7.4 Hz, 2H), 7.32 (dd, *J* = 16.6, 7.6 Hz, 4H), 7.14 (d, *J* = 8.1 Hz, 3H), 2.53 (dd, *J* = 14.9, 8.0 Hz, 2H), 1.15 (t, *J* = 7.2 Hz, 3H). 13C NMR (63 MHz, DMSO) δ 180.04, 140.47, 140.00, 137.47, 128.82, 128.15, 124.74, 124.30, 124.02, 28.10, 16.09.

*1-Phenyl-3-(o-tolyl)thiourea (3f)*

IR (KBr): 3337, 3141 cm^-1^. ^1^H NMR (250 MHz, DMSO) δ 9.73 (s, 1H), 9.28 (d, *J* = 45.1 Hz, 1H), 7.50 (d, *J* = 6.3 Hz, 2H), 7.26 (dd, *J* = 15.4, 8.4 Hz, 4H), 7.16 (s, 2H), 2.24 (s, 3H). ^13^C NMR (63 MHz, DMSO) δ 180.87, 140.00, 138.26, 135.23, 130.77, 128.86, 128.46, 126.88, 126.53, 124.81, 124.12, 18.34.

*1-(2-Iodophenyl)-3-phenylthiourea (3g)*

IR (KBr): 3294, 3172 cm^-1^. ^1^H NMR (250 MHz, DMSO) δ 10.07 (s, 1H), 9.41 (s, 1H), 7.86 (s, 1H), 7.56 (s, 1H), 7.39 (s, 1H), 7.33 (s, 1H), 7.12 (s, 1H), 7.00 (s, 1H). ^13^C NMR (63 MHz, DMSO) δ 180.67, 140.12, 139.15, 130.35, 129.75, 129.47, 128.93, 126.38, 124.07, 122.79, 121.58, 119.43, 118.48.

*1-(2-Bromophenyl)-3-phenylthiourea (3h)*

IR (KBr): 3296, 3186 cm^-1^. ^1^H NMR (250 MHz, DMSO) δ 10.12 (s, 1H), 9.45 (s, 1H), 7.66 (s, 2H), 7.54 (s, 2H), 7.33 (s, 3H), 7.15 (s, 1H). ^13^C NMR (63 MHz, DMSO) δ 180.72, 139.64, 138.29, 132.97, 130.67, 128.97, 128.31, 128.17, 125.10, 124.08, 121.60.

*1-(4-Chlorophenyl)-3-phenylthiourea (3i)*

IR (KBr): 3419, 3207 cm^-1^. ^1^H NMR (250 MHz, DMSO) δ 9.95 (s, 2H), 7.57 – 7.42 (m, 4H), 7.33 (dd, *J* = 14.3, 7.9 Hz, 4H), 7.11 (t, *J* = 6.9 Hz, 1H), ^13^C NMR (63 MHz, DMSO) δ 180.07, 139.28, 138.24, 128.92, 128.71, 125.63, 125.00, 124.09.

*1-(4-Bromophenyl)-3-phenylthiourea (3j)*

IR (KBr): 3221, 3030 cm^-1^. ^1^H NMR (250 MHz, DMSO) δ 9.94 (s, 2H), 7.62 (s, 1H), 7.48 (s, 5H), 7.33 (d, *J* = 6.8 Hz, 2H), 7.12 (d, *J* = 6.3 Hz, 1H). ^13^C NMR (63 MHz, DMSO) δ 180.03, 139.74, 131.62, 128.91, 125.93, 124.10.

*1-(4-Fluorophenyl)-3-phenylthiourea (3k)*

IR (KBr): 3426, 3213 cm^-1^. ^1^H NMR (250 MHz, DMSO) δ 9.81 (d, *J* = 11.9 Hz, 2H), 7.47 (s, 4H), 7.31 (s, 2H), 7.14 (s, 2H). ^13^C NMR (63 MHz, DMSO) δ 180.46, 161.52, 157.68, 139.82, 136.20, 128.90, 126.69, 124.93, 124.13, 115.63, 115.27.

*1-Phenyl-3-(pyridin-2-yl)thiourea (3l)*

IR (KBr): 3401, 3214 cm^-1^. ^1^H NMR (250 MHz, DMSO) δ 13.83 (s, 1H), 10.88 (s, 1H), 8.29 (s, 1H), 7.80 (d, *J* = 7.0 Hz, 1H), 7.68 (d, *J* = 7.1 Hz, 2H), 7.36 (d, *J* = 7.1 Hz, 2H), 7.30 – 7.14 (m, 2H), 7.07 (s, 1H). ^13^C NMR (63 MHz, DMSO) δ 178.73, 153.74, 146.00, 139.78, 138.61, 128.93, 125.89, 124.62, 118.61, 113.38.


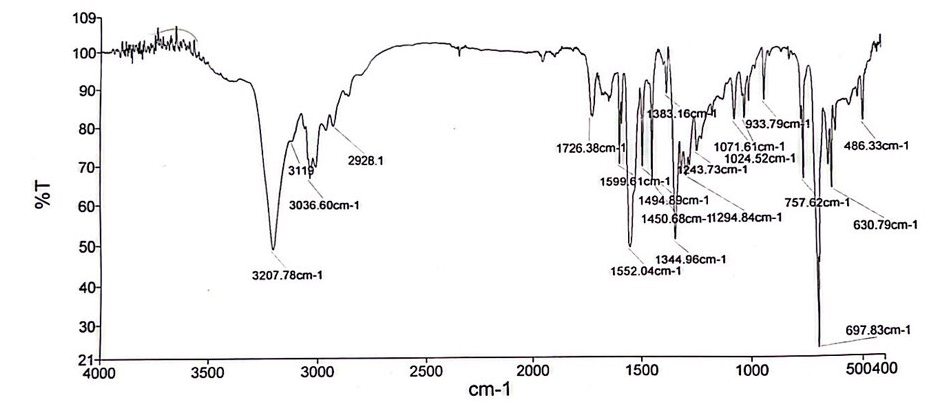


*Figure 2. FTIR Spectrum of 1,3-diphenylthiourea (3a)*

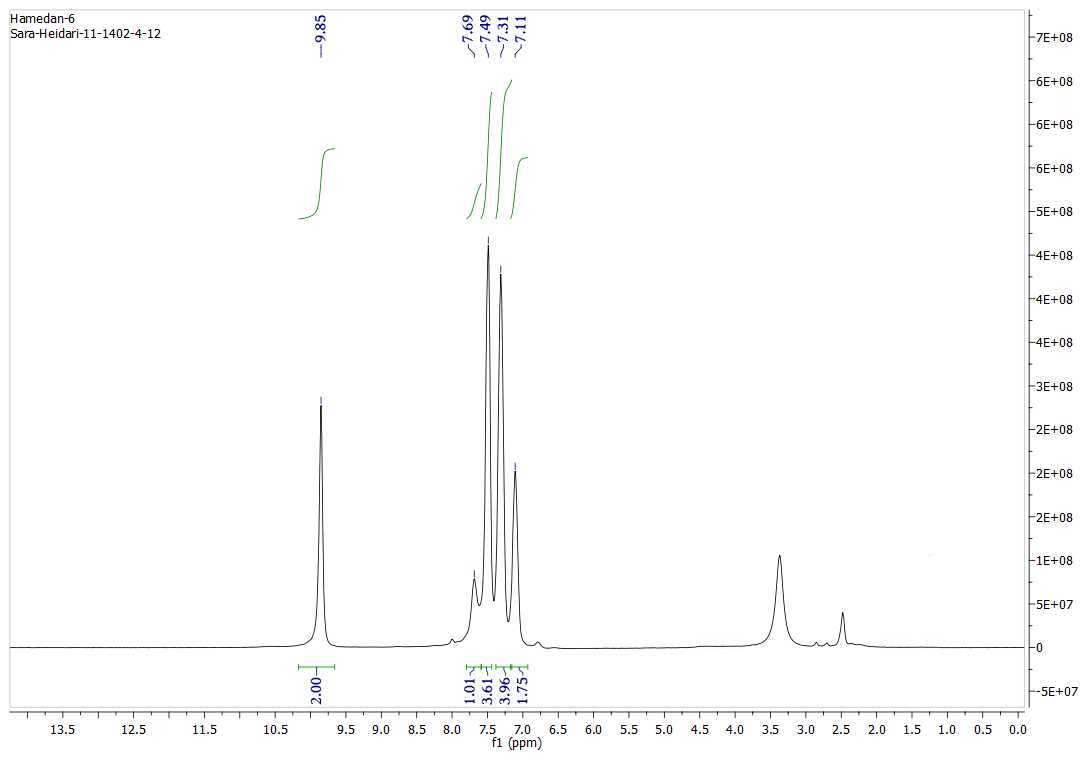


*Figure 3. HNMR Spectrum of 1,3-diphenylthiourea (3a)*


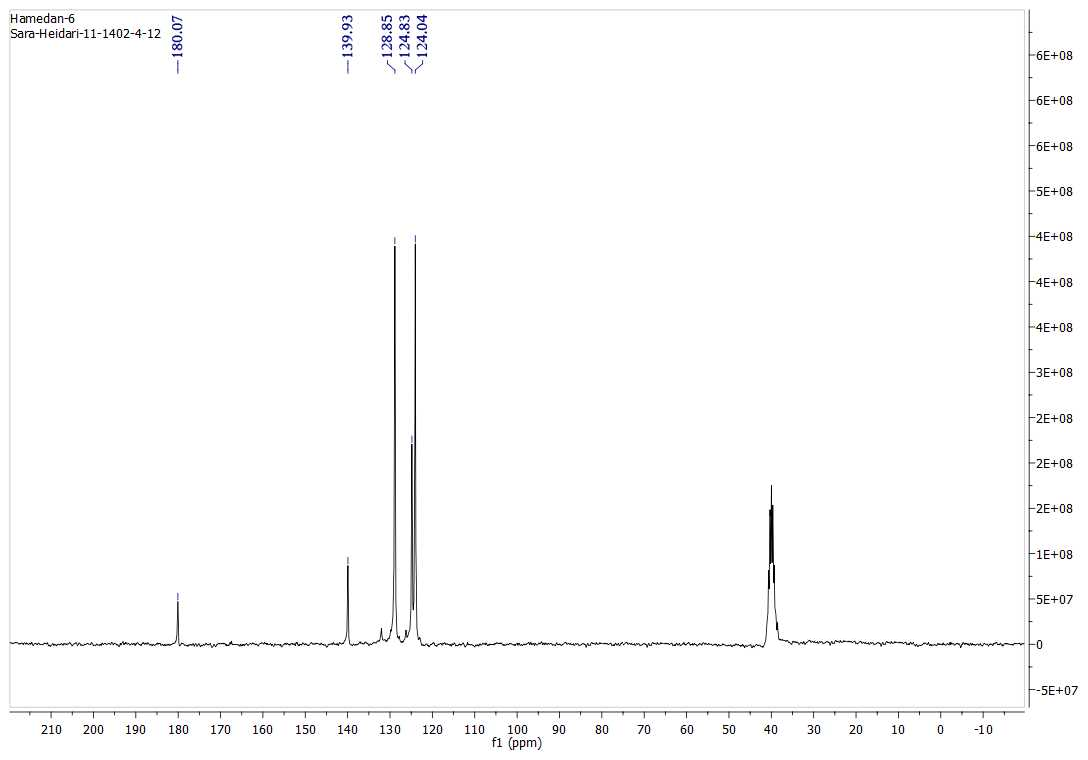


*Figure 4. CNMR Spectrum of 1,3-diphenylthiourea (3a)*


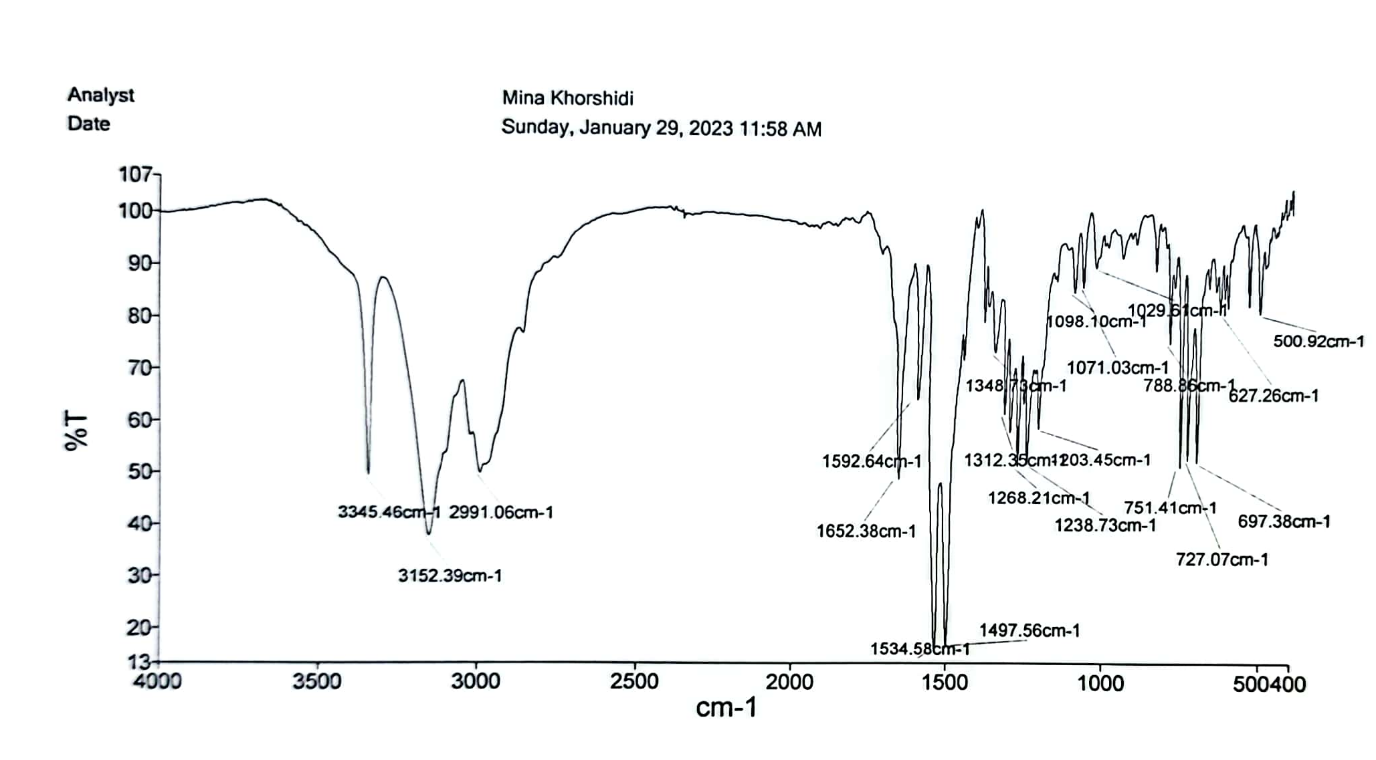


*Figure 5. FTIR Spectrum of 1-(2,3-dimethylphenyl)-3-phenylthiourea (3b)*


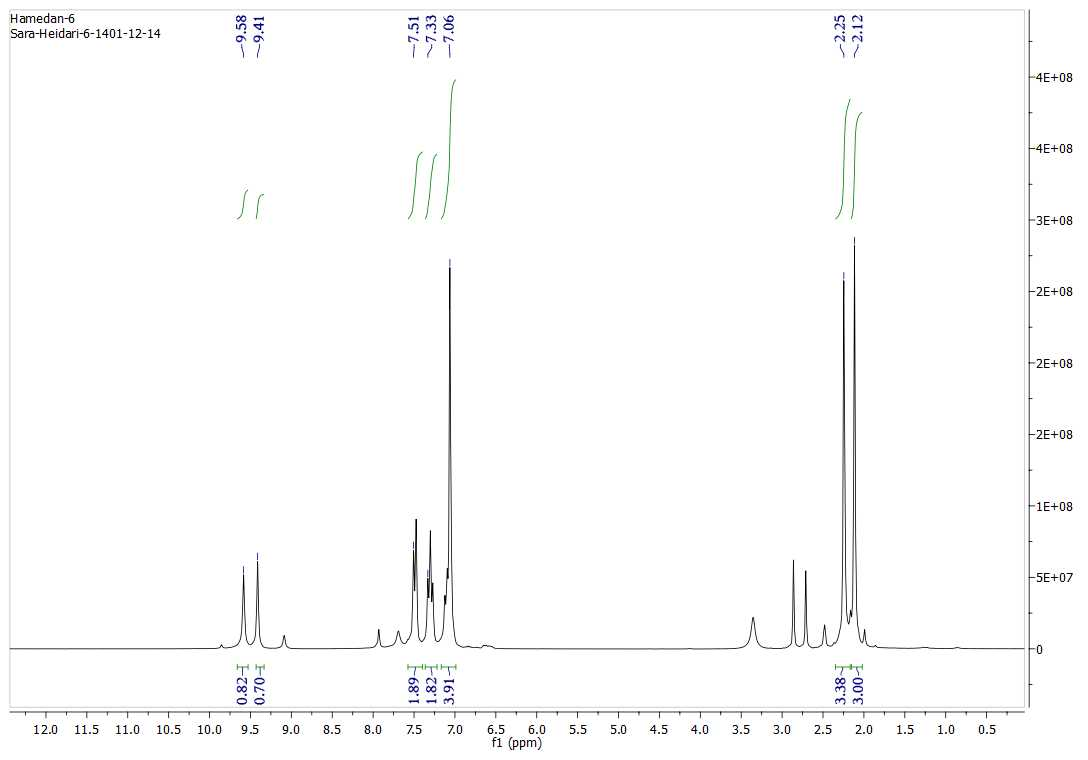


*Figure 6. HNMR Spectrum of 1-(2,3-dimethylphenyl)-3-phenylthiourea (3b)*


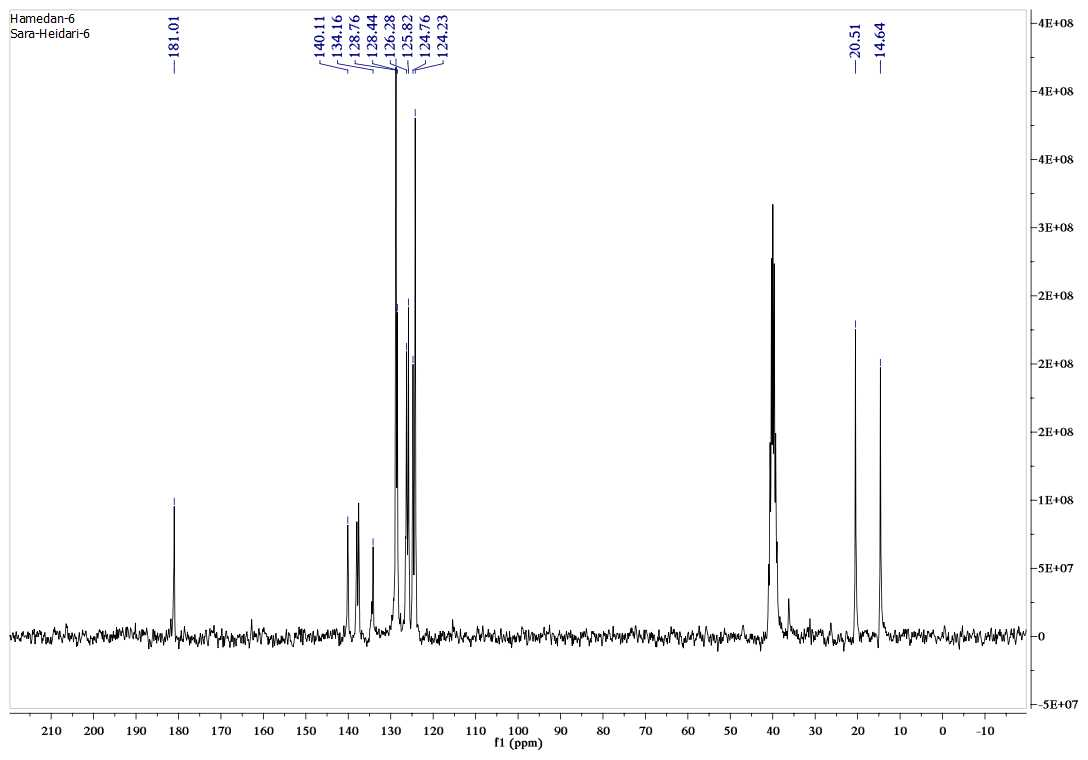


*Figure 7. CNMR Spectrum of 1-(2,3-dimethylphenyl)-3-phenylthiourea (3b*)


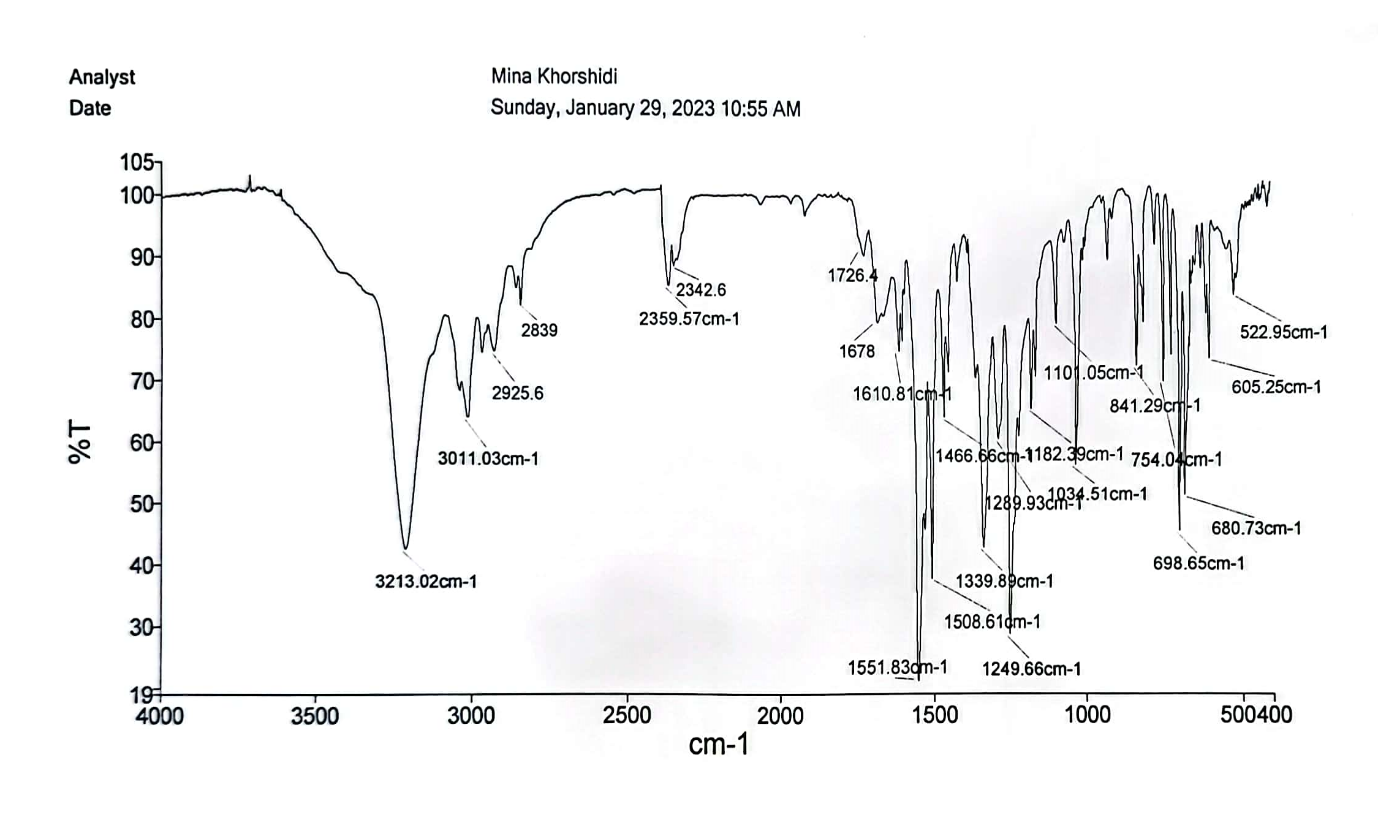


*Figure 8. FTIR Spectrum of 1-(4-methoxyphenyl)-3-phenylthiourea (3c)*


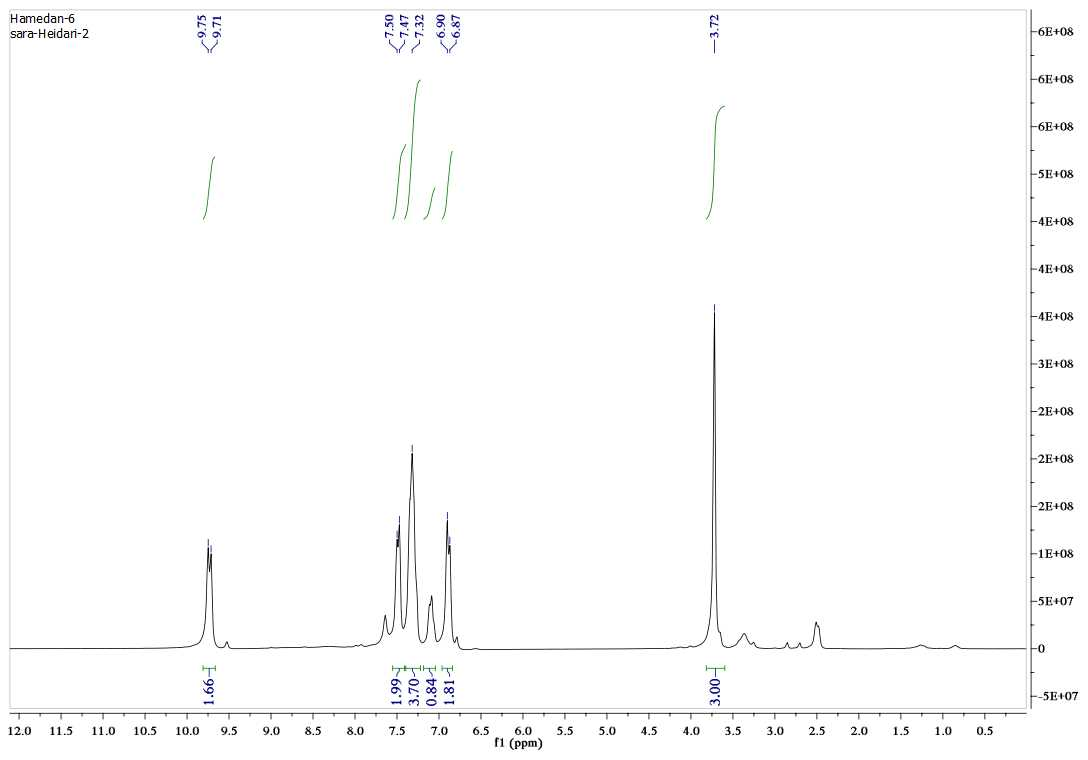


*Figure 9. HNMR Spectrum of 1-(4-methoxyphenyl)-3-phenylthiourea (3c)*


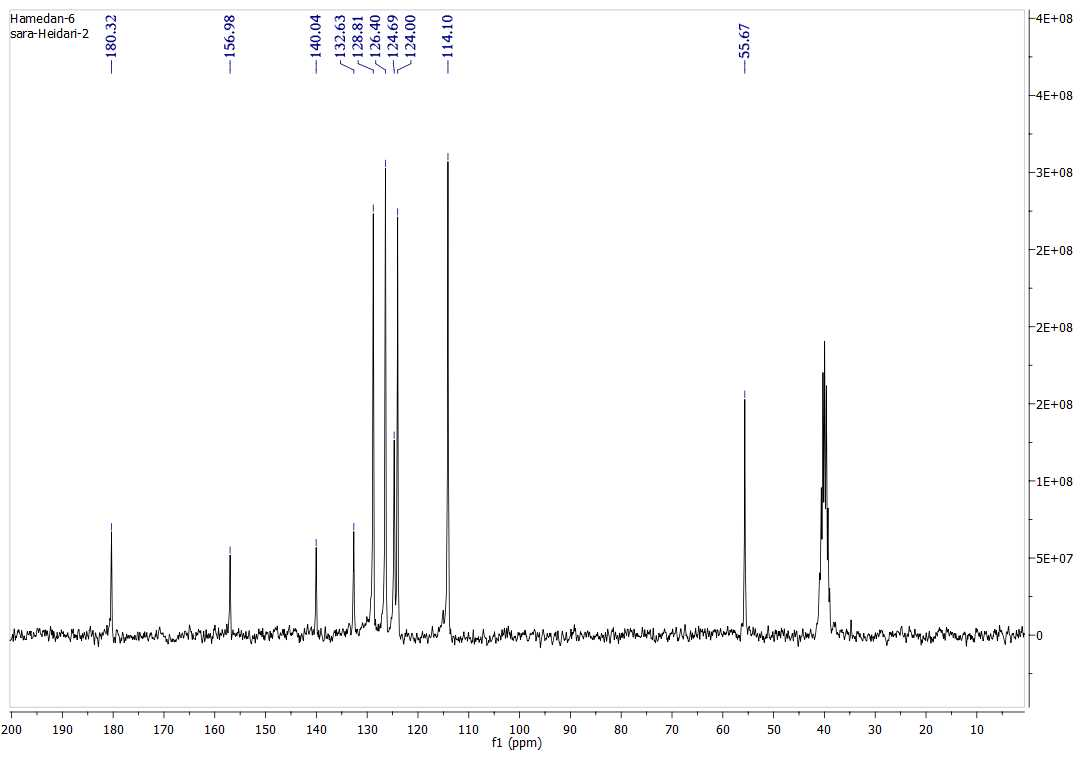


*Figure 10. CNMR Spectrum of 1-(4-methoxyphenyl)-3-phenylthiourea (3c)*


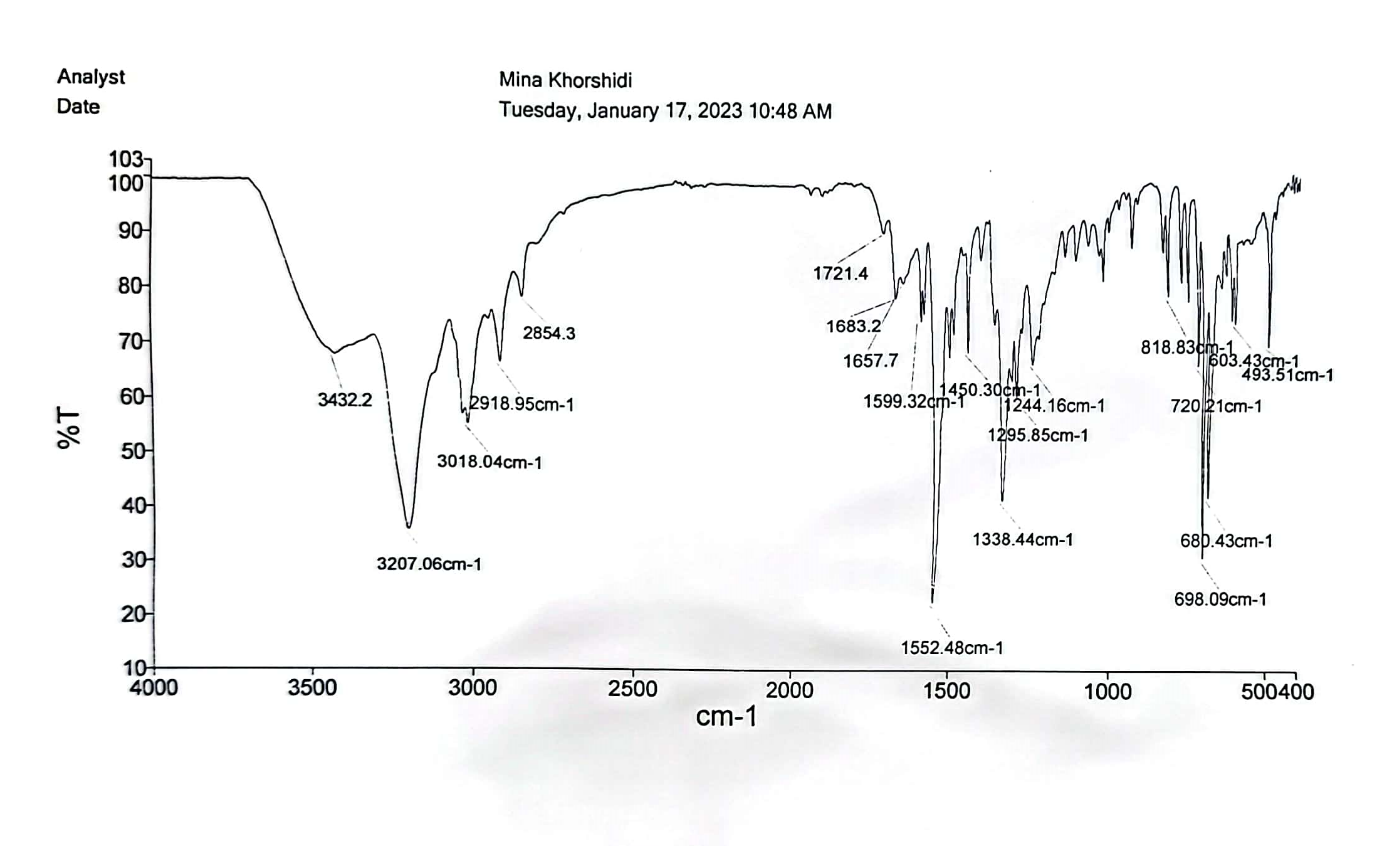


*Figure 11. FTIR Spectrum of 1-phenyl-3-(p-tolyl)thiourea (3d)*


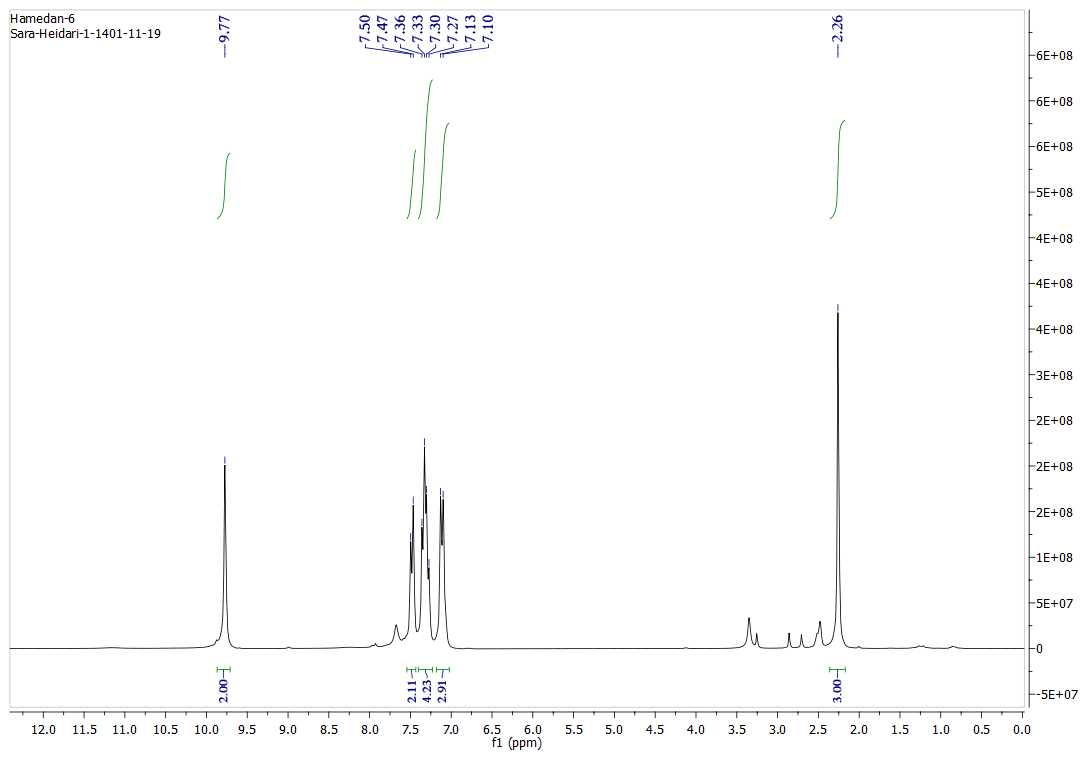


*Figure 12. HNMR Spectrum of 1-phenyl-3-(p-tolyl)thiourea (3d)*


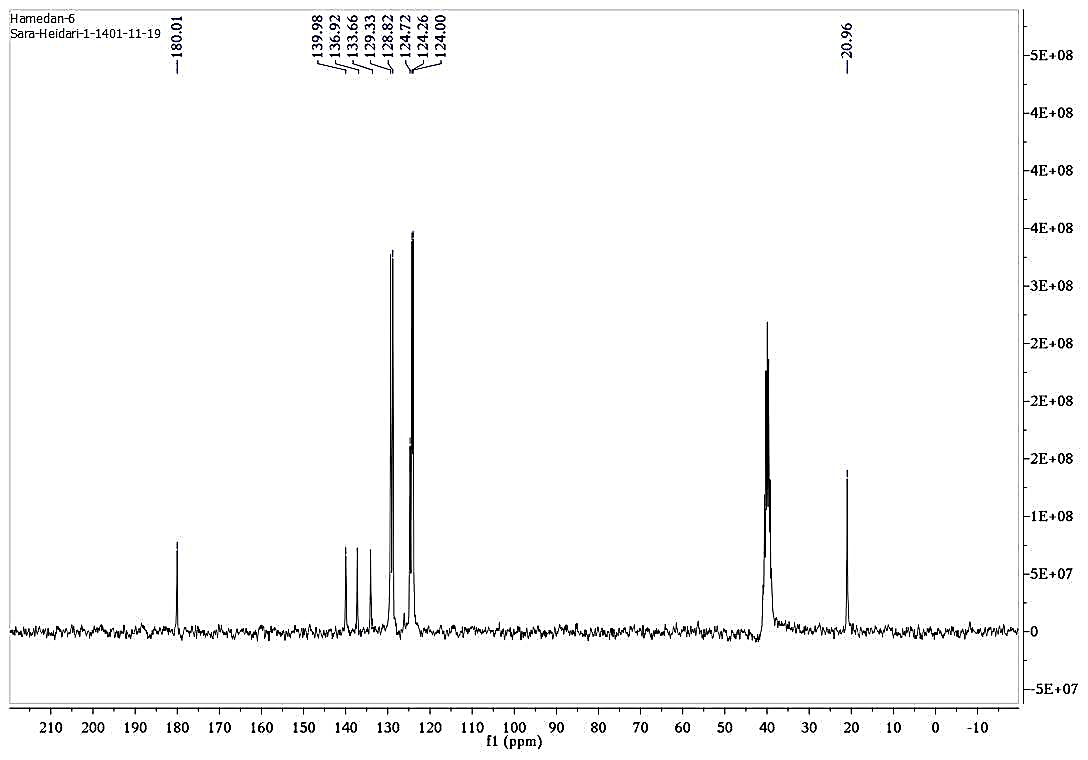


*Figure 13. CNMR Spectrum of 1-phenyl-3-(p-tolyl)thiourea (3d)*

*
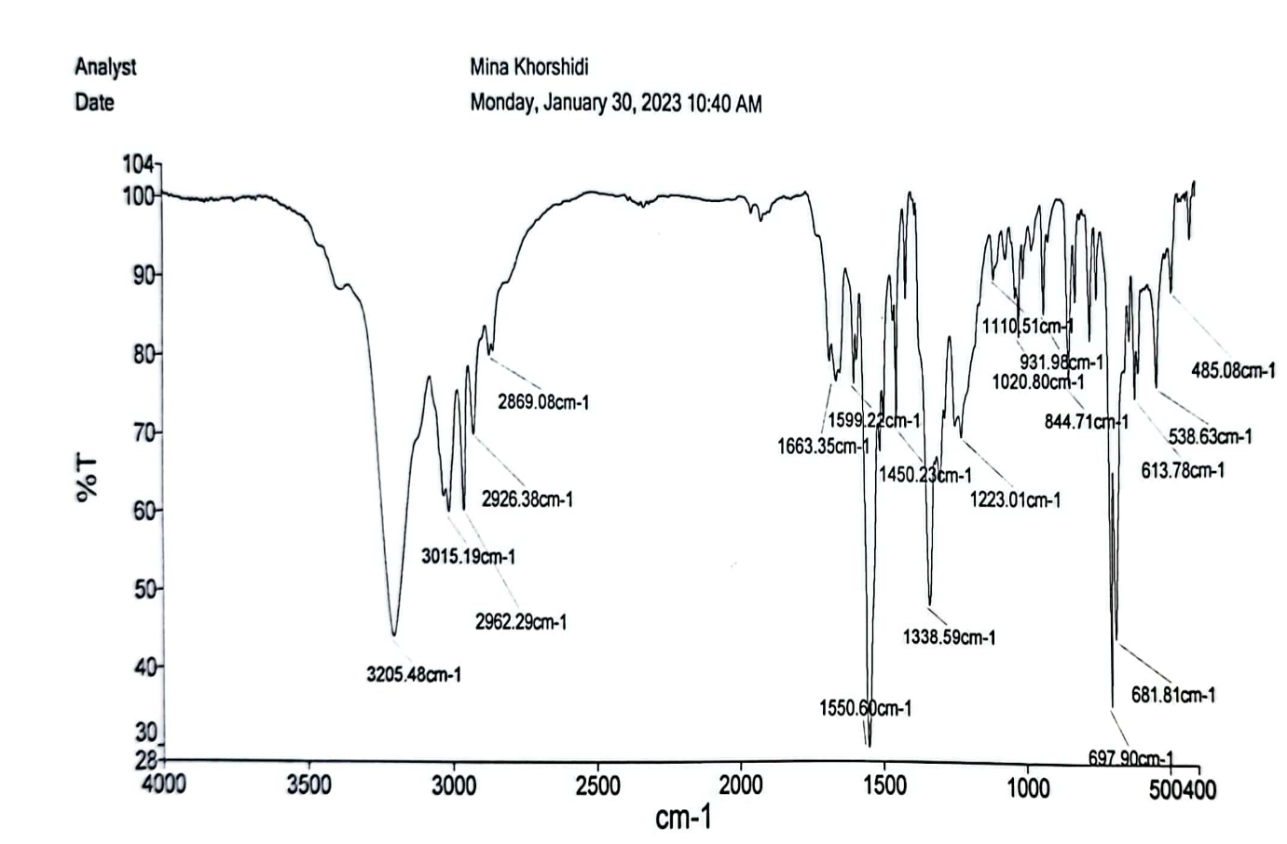
Figure 14. FTIR Spectrum of 1-(4-ethylphenyl)-3-phenylthiourea (3e)*

*****
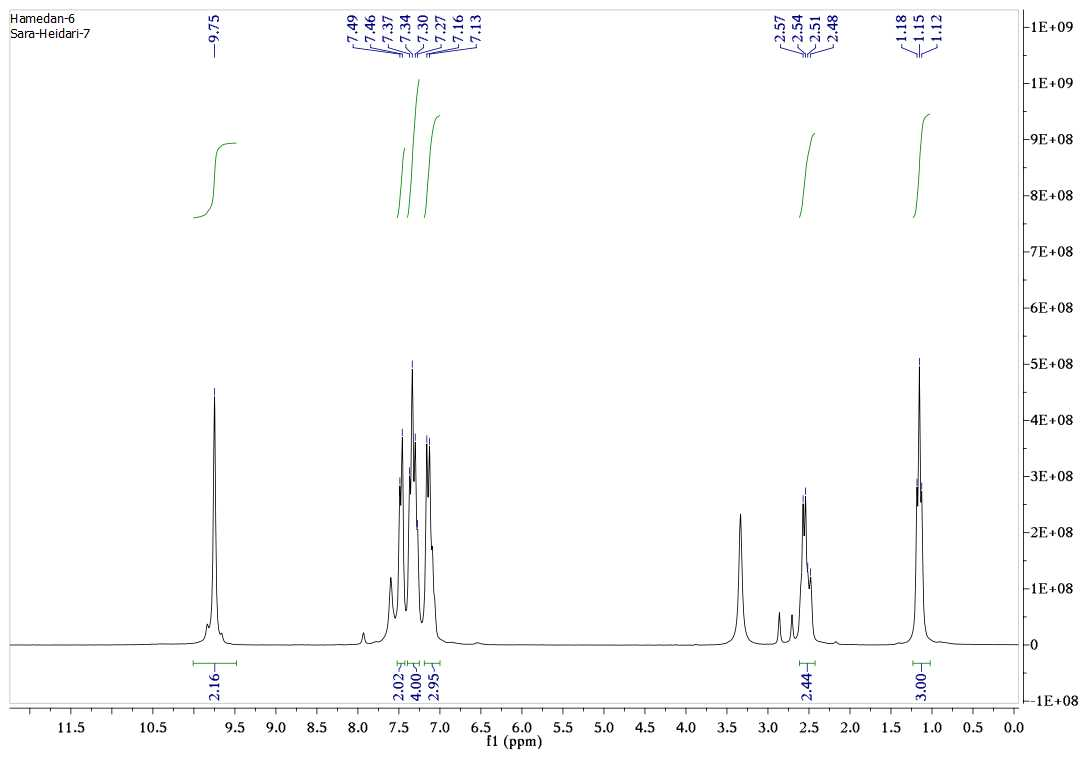
***

*Figure 15. HNMR Spectrum of 1-(4-ethylphenyl)-3-phenylthiourea (3e)*

*****
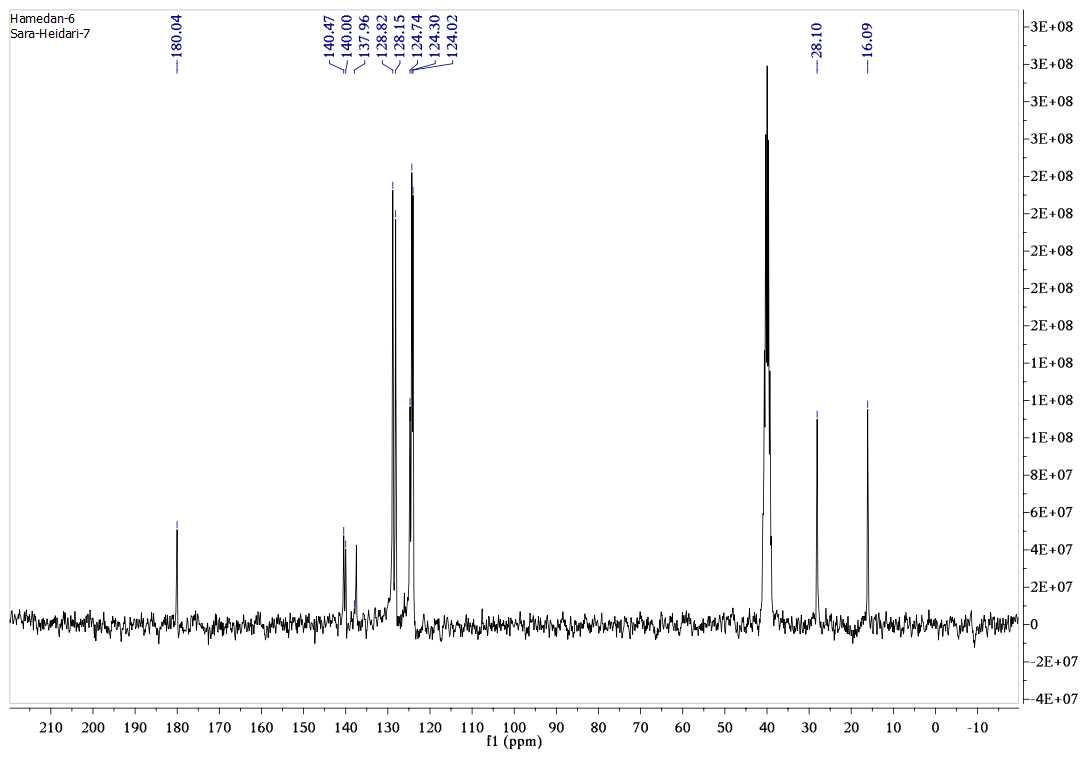
***

*Figure 16. CNMR Spectrum of 1-(4-ethylphenyl)-3-phenylthiourea (3e)*

^^
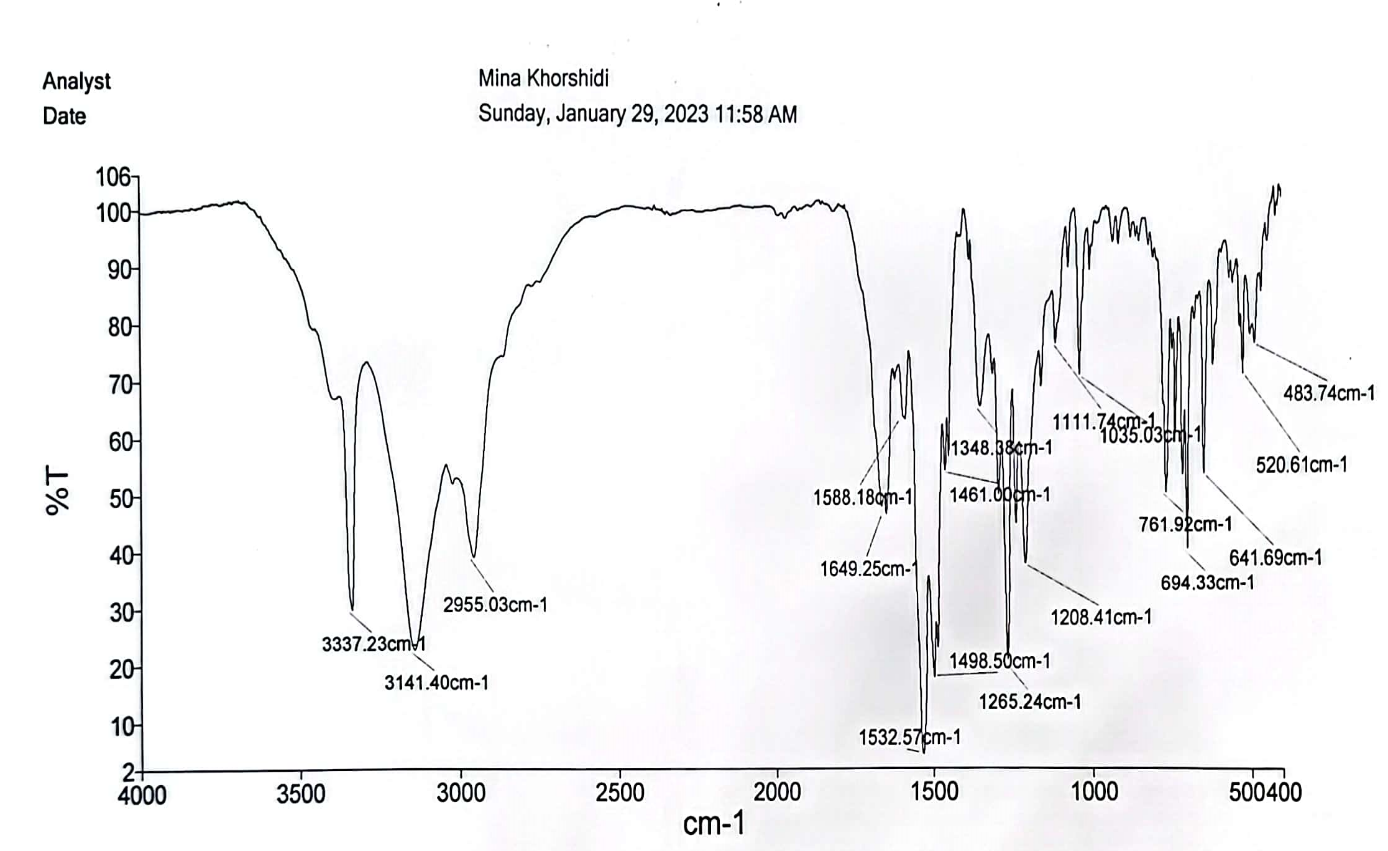


*Figure 17. FTIR Spectrum of 1-Phenyl-3-(o-tolyl)thiourea (3f)*


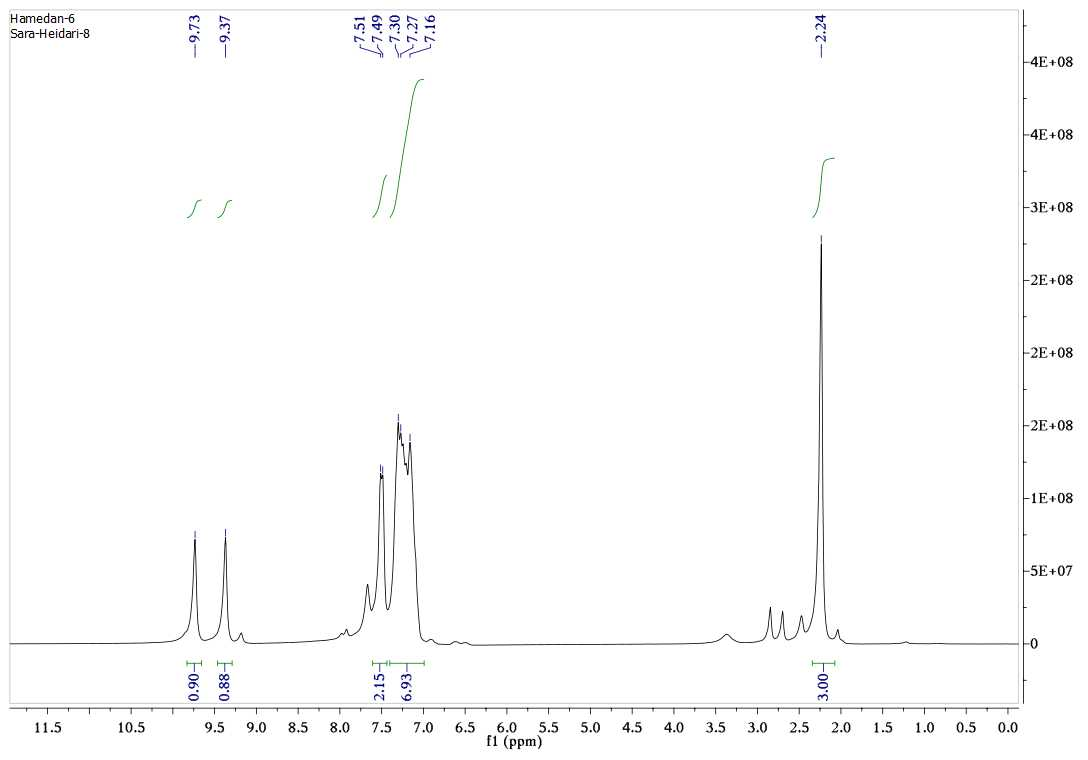


*Figure 18. HNMR Spectrum of 1-phenyl-3-(o-tolyl)thiourea (3f)*


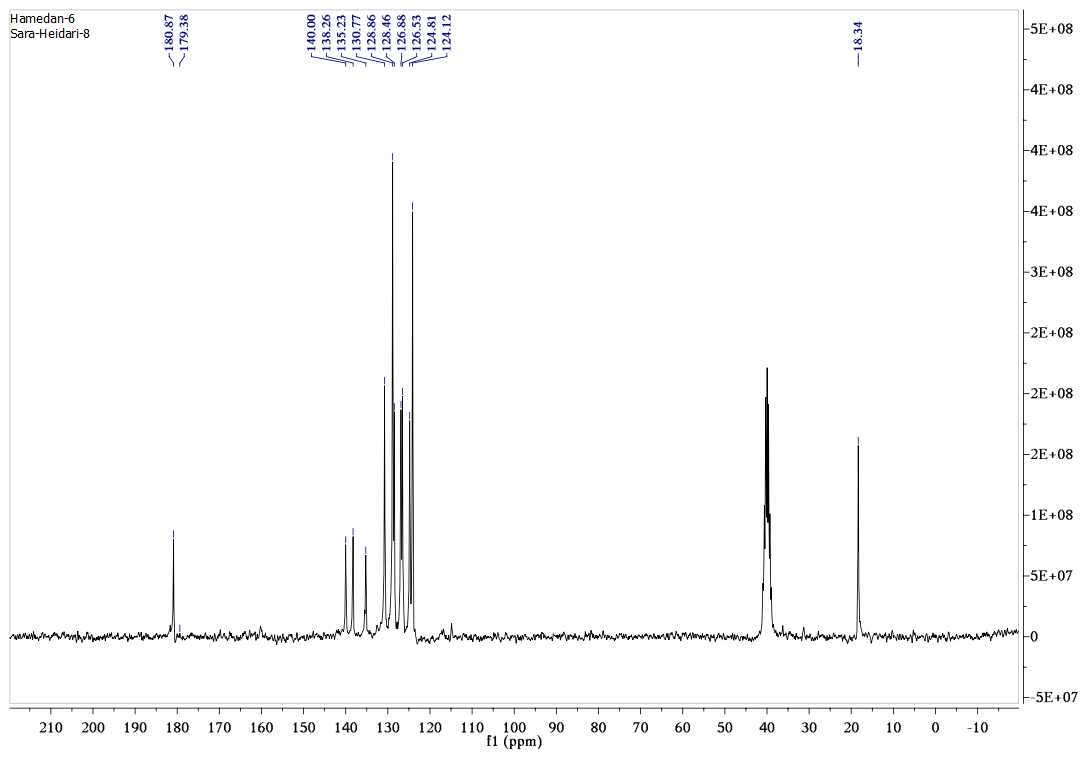


*Figure 19. CNMR Spectrum of 1-phenyl-3-(o-tolyl)thiourea (3f)*


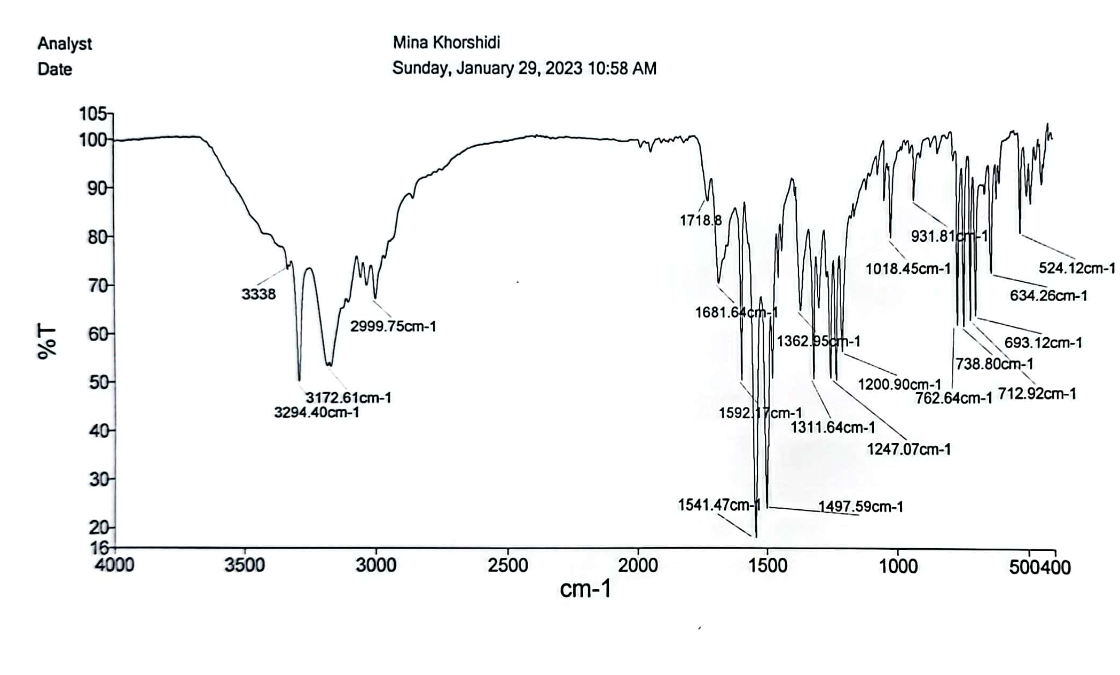


*Figure 20. FTIR Spectrum of 1-(2-iodophenyl)-3-phenylthiourea (3g)*


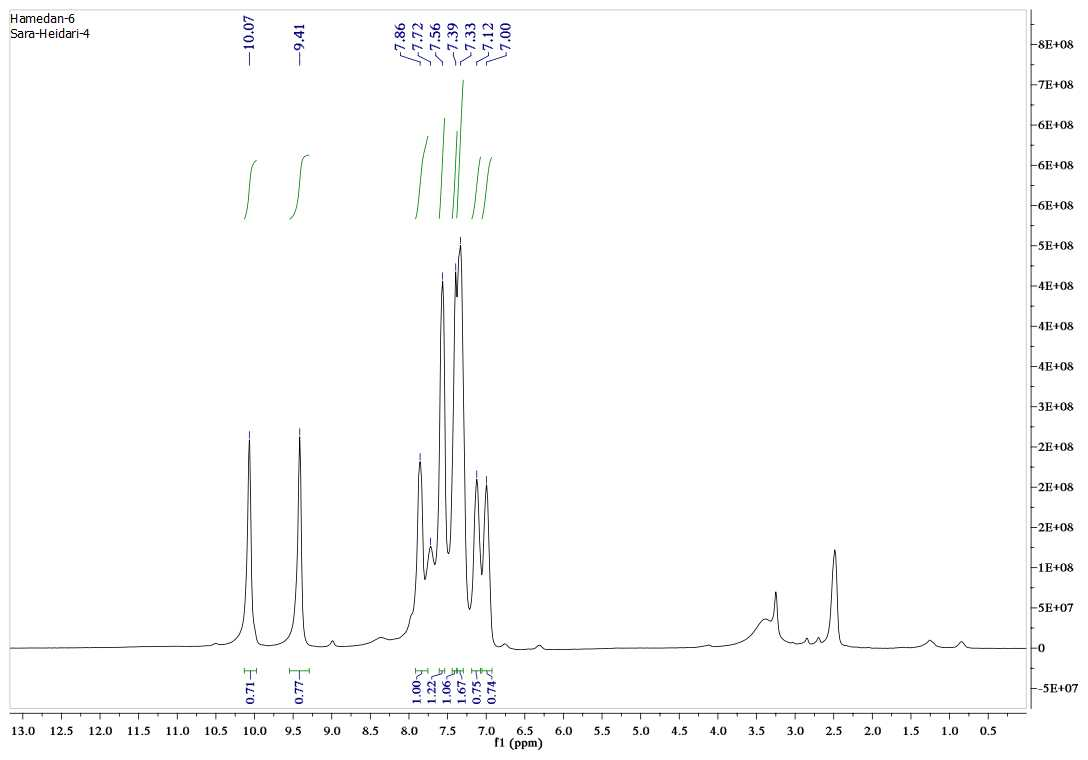


*Figure 21. HNMR Spectrum of 1-(2-iodophenyl)-3-phenylthiourea (3g)*


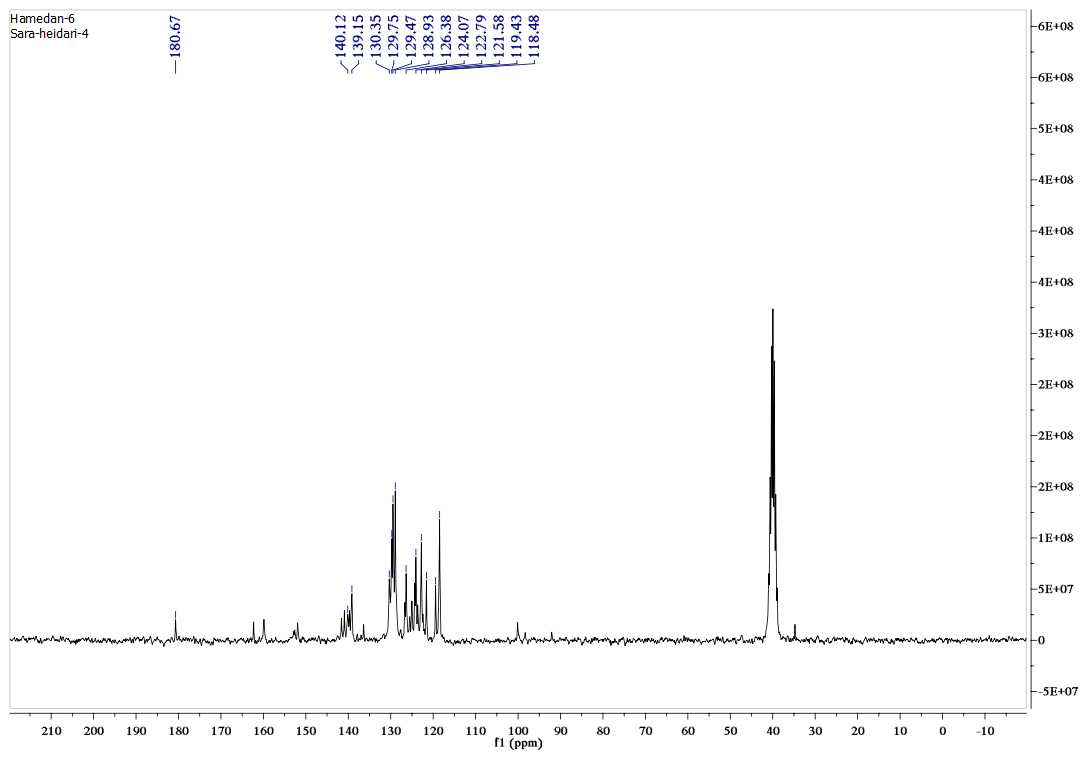


*Figure 22. CNMR Spectrum of* *1-(2-iodophenyl)-3-phenylthiourea (3g)*


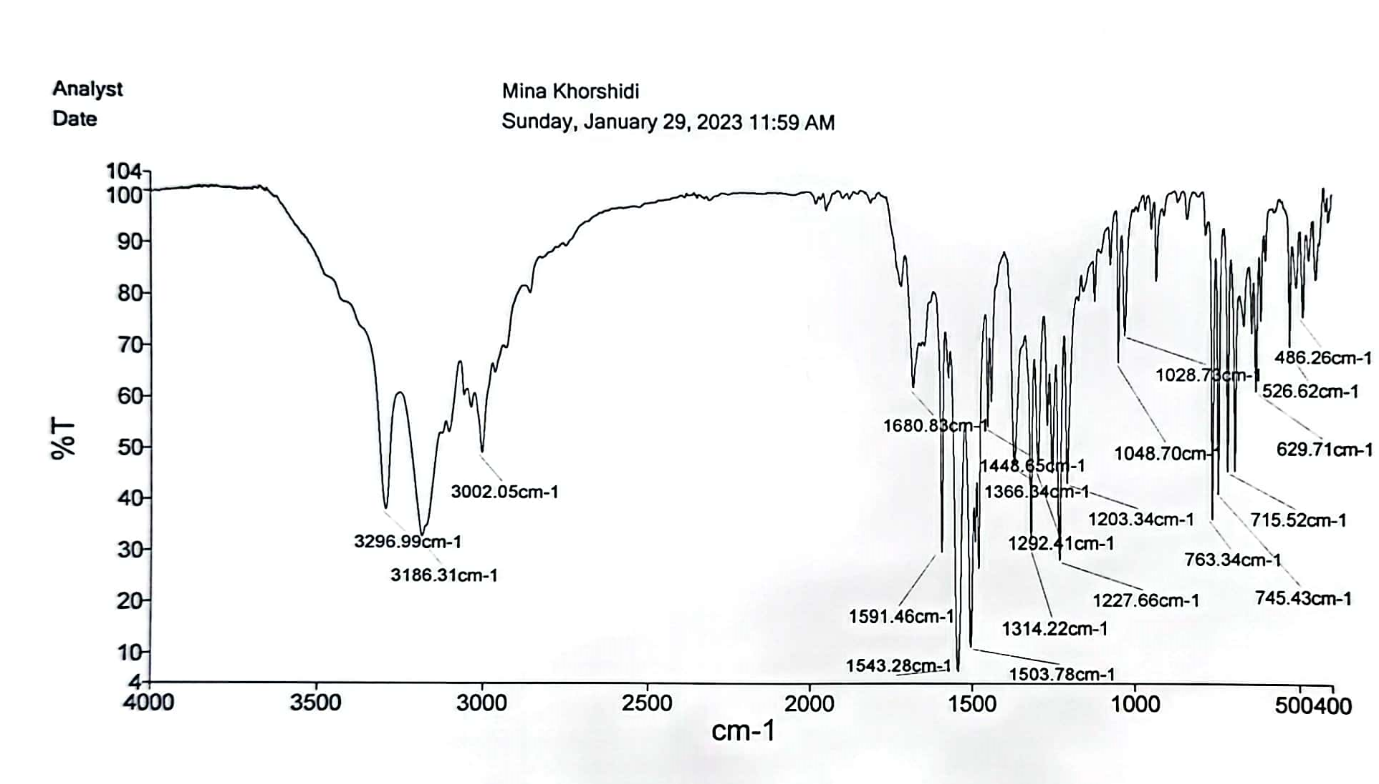


*Figure 23. FTIR Spectrum of 1-(2-bromophenyl)-3-phenylthiourea (3h)*

**
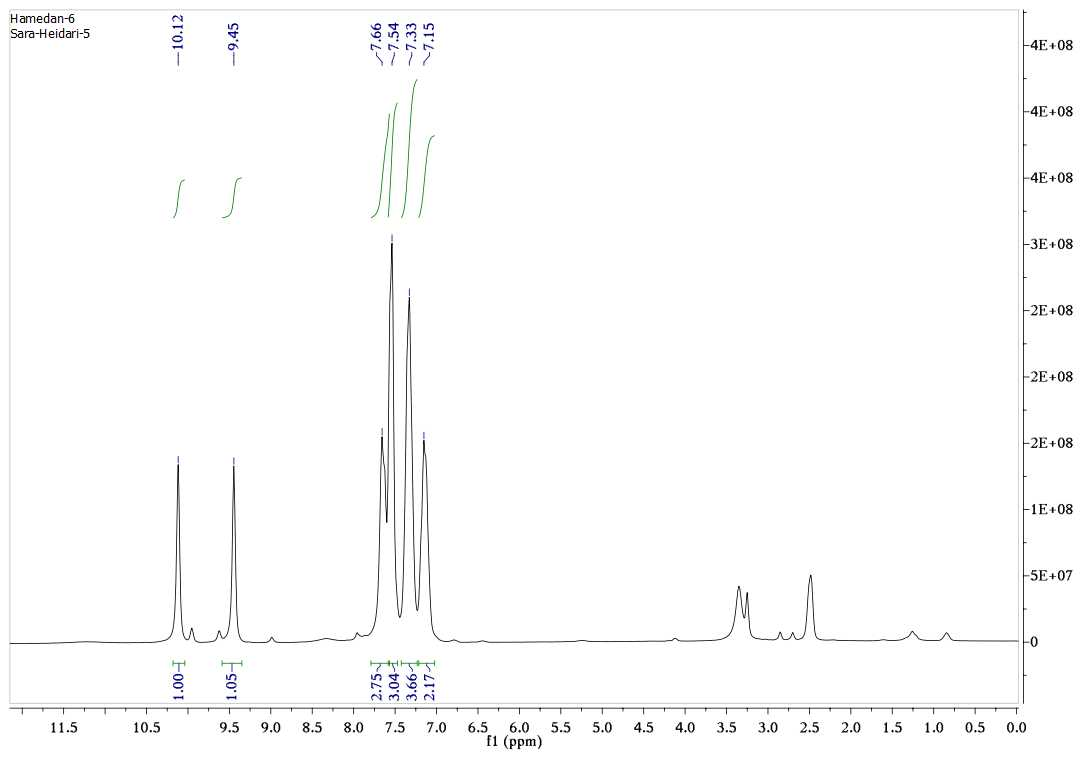
**

*Figure 24. HNMR Spectrum of 1-(2-bromophenyl)-3-phenylthiourea (3h)*

**
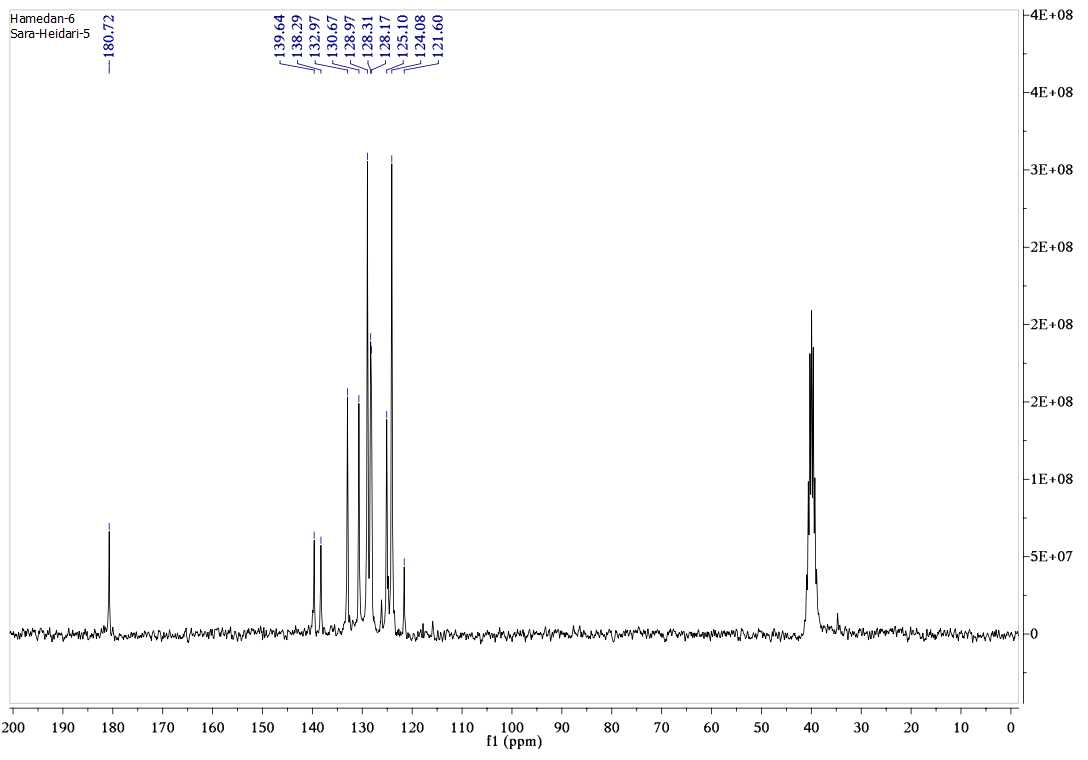
**

*Figure 25. CNMR Spectrum of 1-(2-bromophenyl)-3-phenylthiourea (3h)*

.

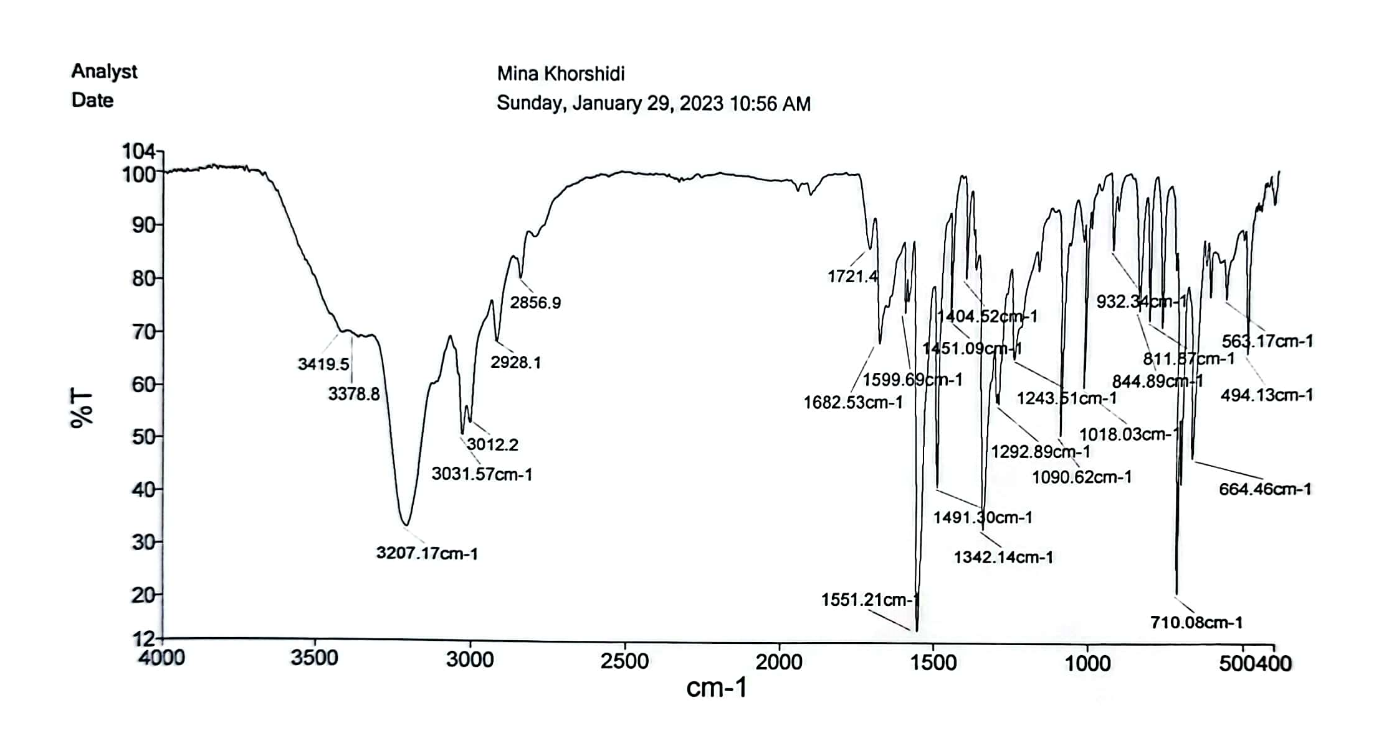


*Figure 26. FTIR Spectrum of 1-(4-chlorophenyl)-3-phenylthiourea (3i)*


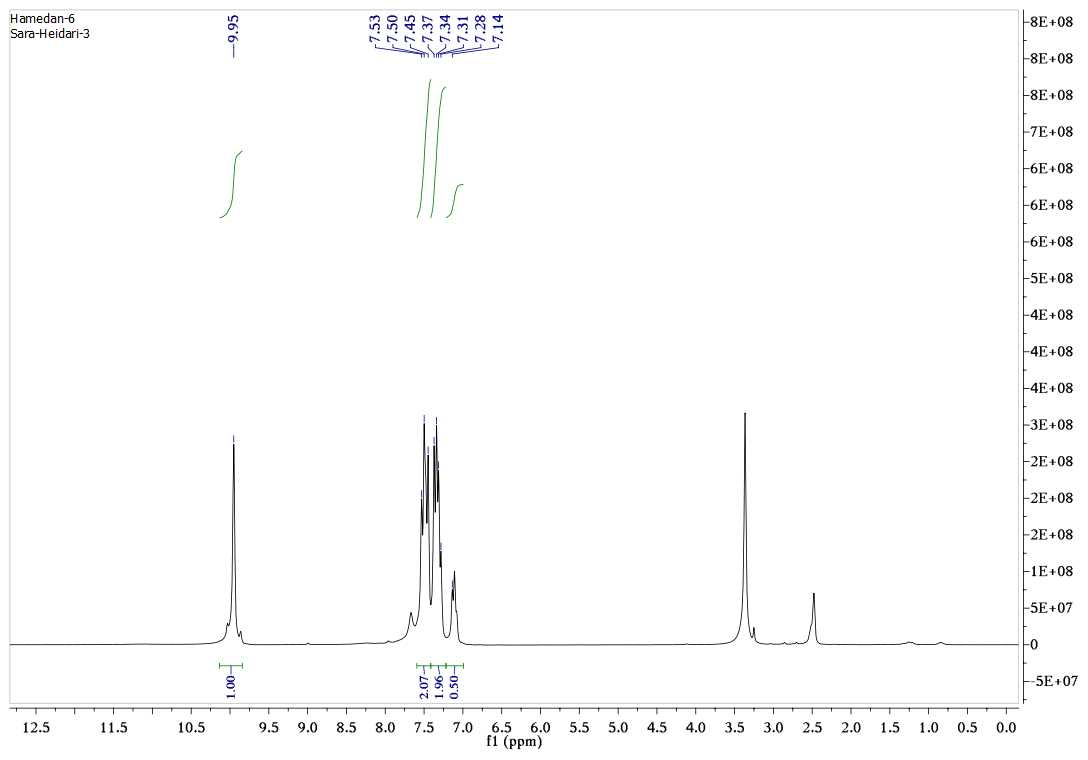


*Figure 27. HNMR Spectrum of 1-(4-chlorophenyl)-3-phenylthiourea (3i)*


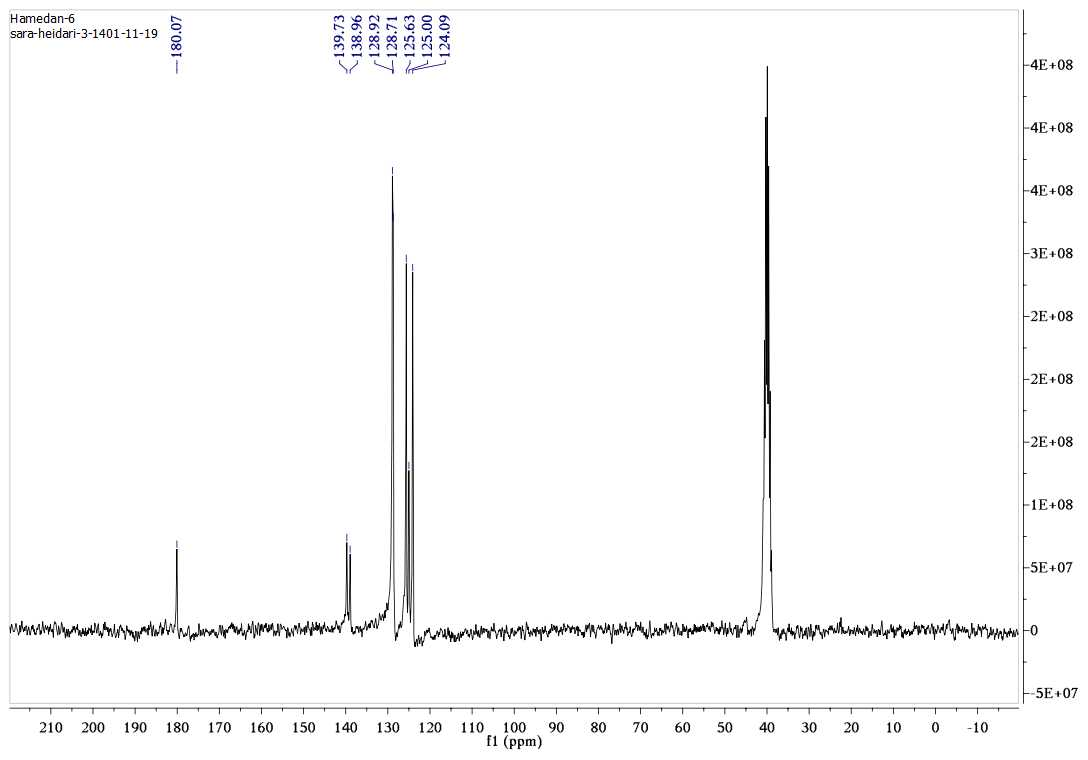


*Figure 28. CNMR Spectrum of 1-(4-chlorophenyl)-3-phenylthiourea (3i)*


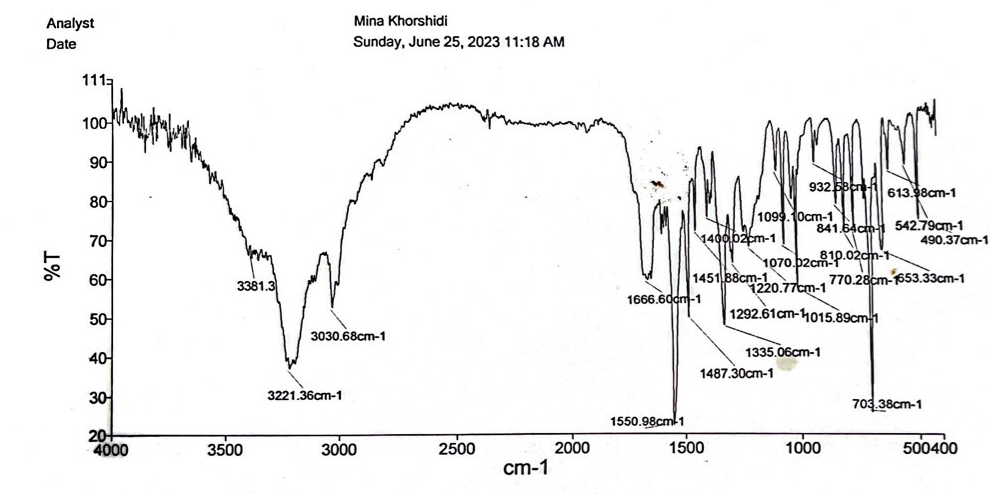


*Figure 29. FTIR Spectrum of 1-(4-Bromophenyl)-3-phenylthiourea (3j)*


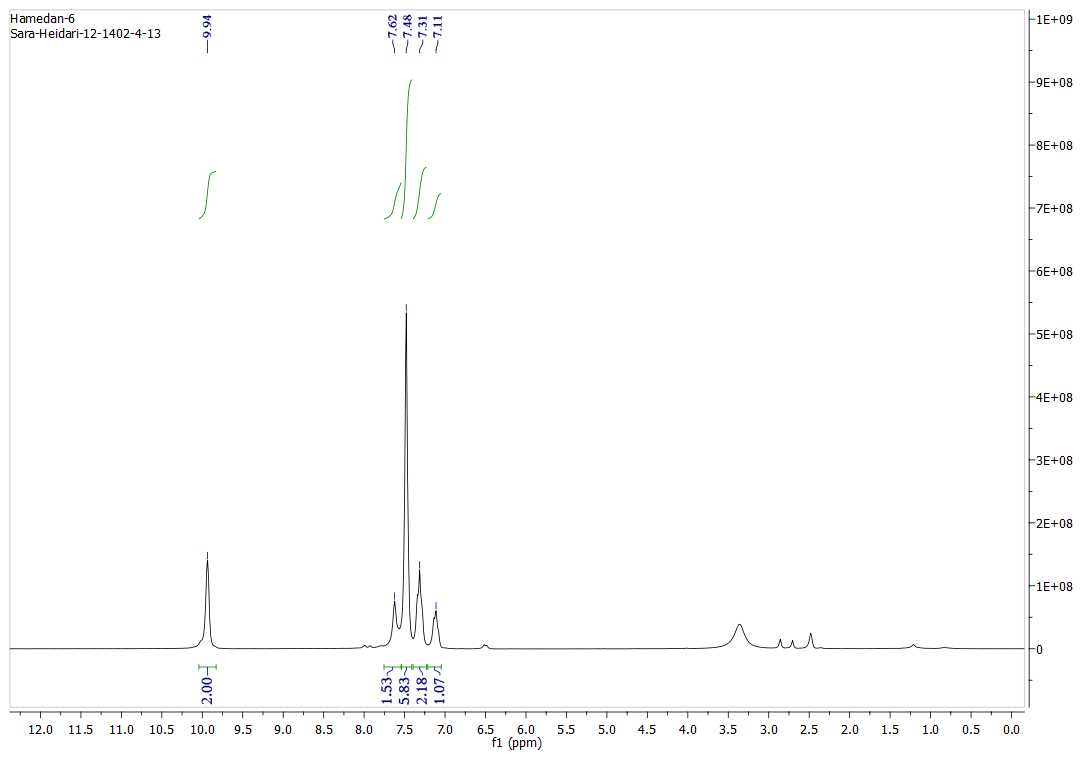


*Figure 30. HNMR Spectrum of 1-(4-bromophenyl)-3-phenylthiourea (3j)*


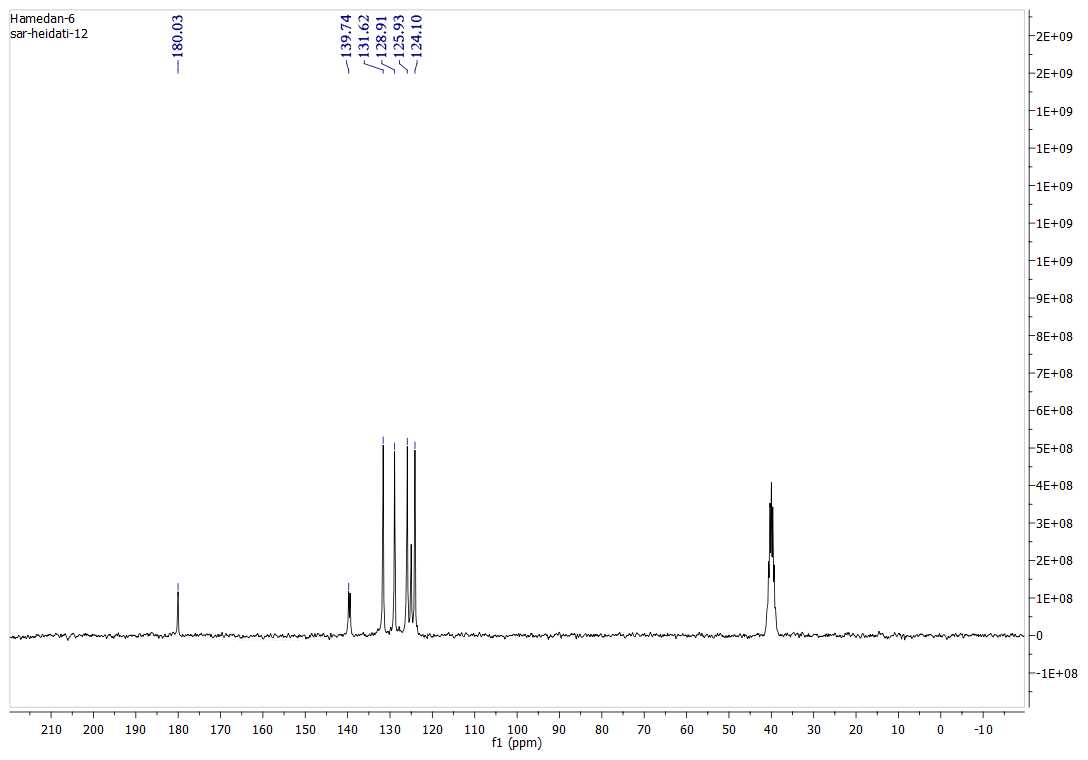


*Figure 31. CNMR Spectrum of 1-(4-bromophenyl)-3-phenylthiourea (3j)*


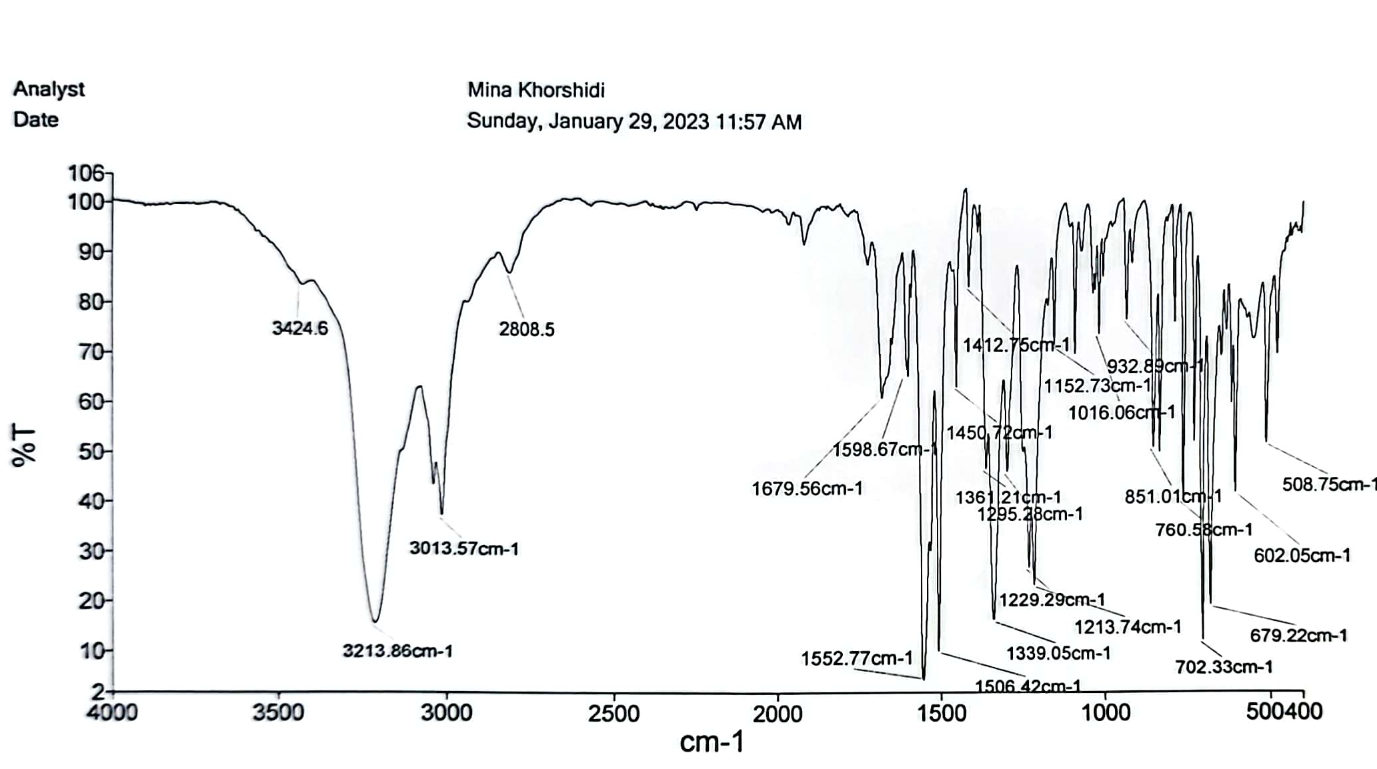


*Figure 32. FTIR Spectrum of 1-(4-fluorophenyl)-3-phenylthiourea (3k)*


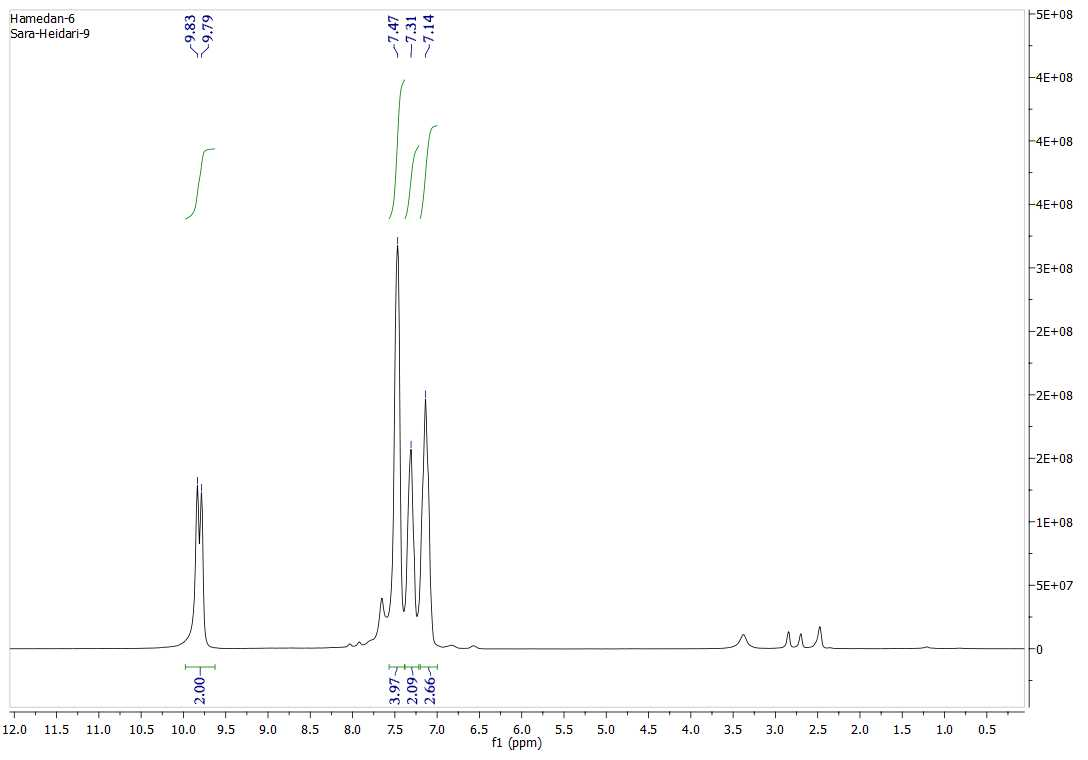


*Figure 33. HNMR Spectrum of 1-(4-fluorophenyl)-3-phenylthiourea (3k)*


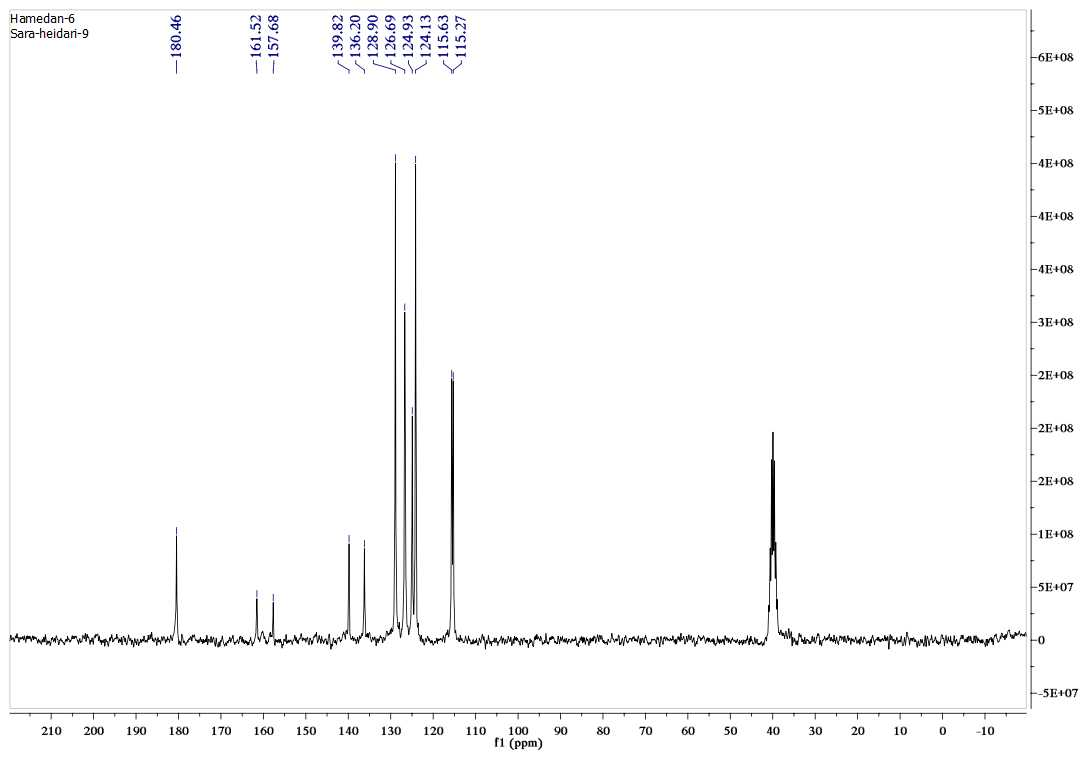


*Figure 34. CNMR Spectrum of 1-(4-fluorophenyl)-3-phenylthiourea (3k)*


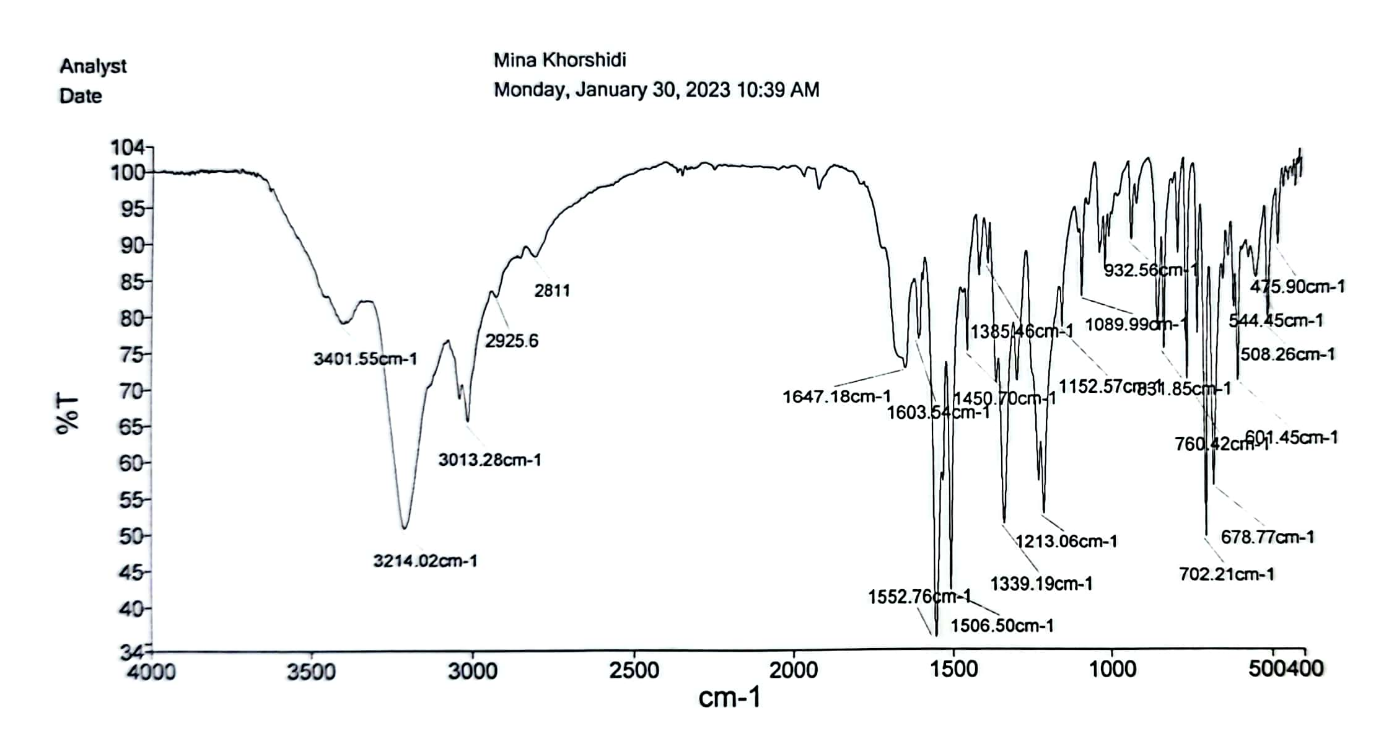


*Figure 35. FTIR Spectrum of 1-phenyl-3-(pyridin-2-yl)thiourea (3l)*


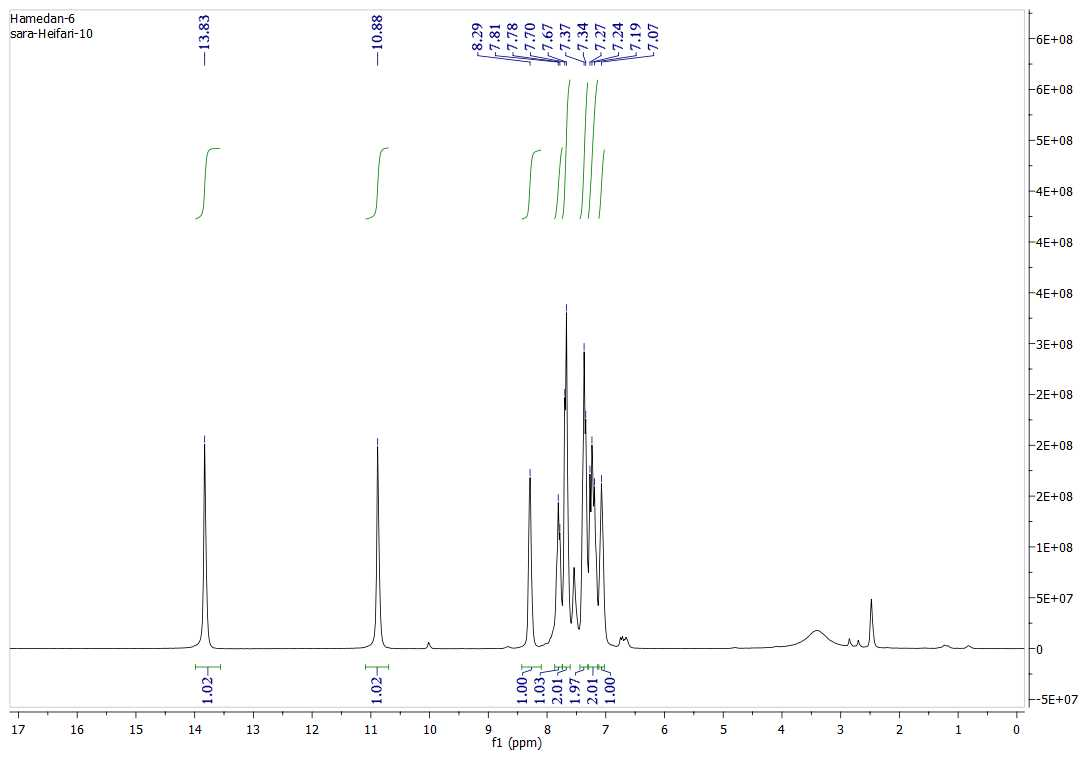


*Figure 36. HNMR Spectrum of 1-phenyl-3-(pyridin-2-yl)thiourea (3l)*


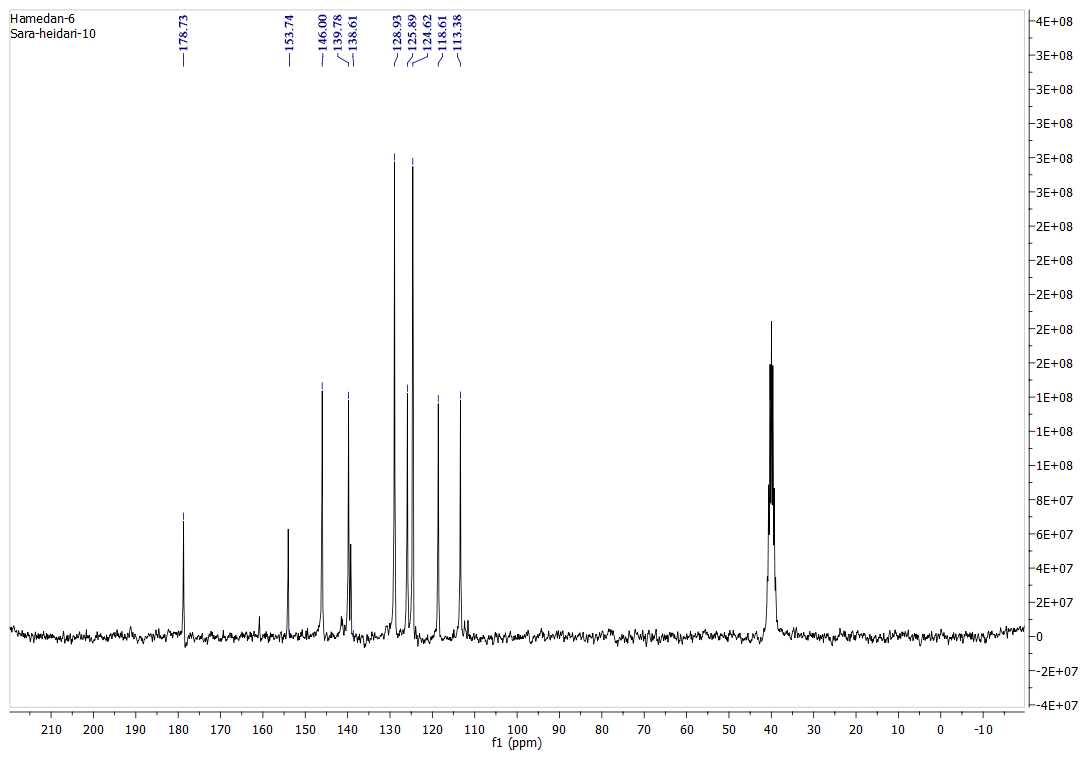


*Figure 37. CNMR Spectrum of 1-phenyl-3-(pyridin-2-yl)thiourea (3l)*
